# Achieving a “step change” in the tuberculosis epidemic through comprehensive community-wide intervention: a model-based analysis

**DOI:** 10.1186/s12916-021-02110-5

**Published:** 2021-10-14

**Authors:** Sourya Shrestha, Emily A. Kendall, Rebekah Chang, Roy Joseph, Parastu Kasaie, Laura Gillini, Anthony Todd Fojo, Michael Campbell, Nimalan Arinaminpathy, David W. Dowdy

**Affiliations:** 1 Department of Epidemiology, Johns Hopkins Bloomberg School of Public Health, Baltimore, MD 21205 USA; 2 Johns Hopkins School of Medicine, Baltimore, USA; 3 Clinton Health Access Initiative, Boston, USA; 4 Department of Infectious Disease Epidemiology, Imperial College, London, UK

**Keywords:** Tuberculosis, India, Tuberculosis modeling, Tuberculosis active case finding, Tuberculosis preventive therapy, Tuberculosis health system strengthening

## Abstract

**Background:**

Global progress towards reducing tuberculosis (TB) incidence and mortality has consistently lagged behind the World Health Organization targets leading to a perception that large reductions in TB burden cannot be achieved. However, several recent and historical trials suggest that intervention efforts that are comprehensive and intensive can have a substantial epidemiological impact. We aimed to quantify the potential epidemiological impact of an intensive but realistic, community-wide campaign utilizing existing tools and designed to achieve a “step change” in the TB burden.

**Methods:**

We developed a compartmental model that resembled TB transmission and epidemiology of a mid-sized city in India, the country with the greatest absolute TB burden worldwide. We modeled the impact of a one-time, community-wide screening campaign, with treatment for TB disease and preventive therapy for latent TB infection (LTBI). This one-time intervention was followed by the strengthening of the tuberculosis-related health system, potentially facilitated by leveraging the one-time campaign. We estimated the tuberculosis cases and deaths that could be averted over 10 years using this comprehensive approach and assessed the contributions of individual components of the intervention.

**Results:**

A campaign that successfully screened 70% of the adult population for active and latent tuberculosis and subsequently reduced diagnostic and treatment delays and unsuccessful treatment outcomes by 50% was projected to avert 7800 (95% range 5450–10,200) cases and 1710 (1290–2180) tuberculosis-related deaths per 1 million population over 10 years. Of the total averted deaths, 33.5% (28.2–38.3) were attributable to the inclusion of preventive therapy and 52.9% (48.4–56.9) to health system strengthening.

**Conclusions:**

A one-time, community-wide mass campaign, comprehensively designed to detect, treat, and prevent tuberculosis with currently existing tools can have a meaningful and long-lasting epidemiological impact. Successful treatment of LTBI is critical to achieving this result. Health system strengthening is essential to any effort to transform the TB response.

**Supplementary Information:**

The online version contains supplementary material available at 10.1186/s12916-021-02110-5.

## Background

Global progress in fighting tuberculosis (TB) has languished for decades. Currently, TB incidence is falling by only 2% per year worldwide, far behind the pace necessary to achieve WHO’s End TB target of a 90% reduction in TB incidence between 2015 and 2035 [1, 2]. Of the eight highest-burden countries that account for two-thirds of new cases, five of them—Indonesia, the Philippines, Pakistan, Nigeria, and Bangladesh—have experienced less than 1% annual decline in incidence over the last decade [1]. The decline exceeded 5% per year only in South Africa, where this decline has been predominantly among HIV-positive individuals [1]. It is likely that progress will continue at this unacceptably slow pace unless a feasible, actionable strategy can be developed that holds a reasonable promise of achieving a “step change” in the TB epidemic, defined for the purposes of this analysis as a significant and sustainable change in the burden of TB effected over a short period of time.

Historical and modern evidence clearly demonstrates that rapid declines in TB burden are possible [3, 4]. For example, a comprehensive campaign to find TB cases and treat latent TB infection (LTBI) in the Yukon-Kuskokwim Delta (Alaska, USA) saw the annual risk of TB infection fall from 24.6% in 1949–1951 to 1.1% in 1960 [3]. More recently, community-wide screening for active TB in Cà Mau Province, Vietnam, reduced prevalence by 44% over 3 years, with potential reductions in transmission and TB incidence to be observed over the coming years [4]. These studies, combined with examples of successful TB control programs implemented at the regional and national levels [5, 6], provide evidence that substantial reductions in TB burden can be achieved with focused, intensive effort; however, scalable and sustainable approaches for achieving such reductions have yet to be developed. Modern tools—including, for example, highly portable digital X-ray devices with emerging artificial intelligence (AI)-based interpretation [7], adoption of novel short-course preventive therapy with drugs whose price is being cut dramatically [8], and rapid high-sensitivity molecular testing for TB (and drug resistance) [9]—could facilitate implementation of intensive, broad-scale efforts to find, treat, and prevent TB that previously might have been deemed infeasible.

To date, modeling efforts to project the impact of TB-focused interventions have tended to focus on individual interventions (e.g., diagnosis with Xpert MTB/RIF [10], LTBI treatment [11], household contact investigation [12]) or achievement of specific elimination goals or other targets [13, 14]. Few studies have attempted to estimate the population-level impact of a comprehensive yet feasible approach to halt TB transmission at the community level. Such broad-scale efforts are intrinsically challenging to scale up, but one attractive approach is a “surge/maintenance” strategy, involving an initial, time-limited phase of high-intensity intervention followed by a more sustained phase of health system strengthening that is facilitated by the initial “surge.”

Here, we aimed to quantify the potential epidemiological impact of an intensive but realistic, community-wide campaign, designed to achieve a “step change” in the context of a high-burden urban population. Our rationale was that, if a realistic campaign could effect significant and sustainable change in a short period of time, it could motivate further innovation, wider adoption, and greater enthusiasm for funding such an approach on a broader scale. We conceptualized the model to represent a medium-sized city in India—the country with the largest number of TB cases (more than 25% of the global burden), containing multiple large cities in which TB-related interventions could be carried out on a city-wide scale.

## Methods

### Model conceptualization

**Fig. 1:**
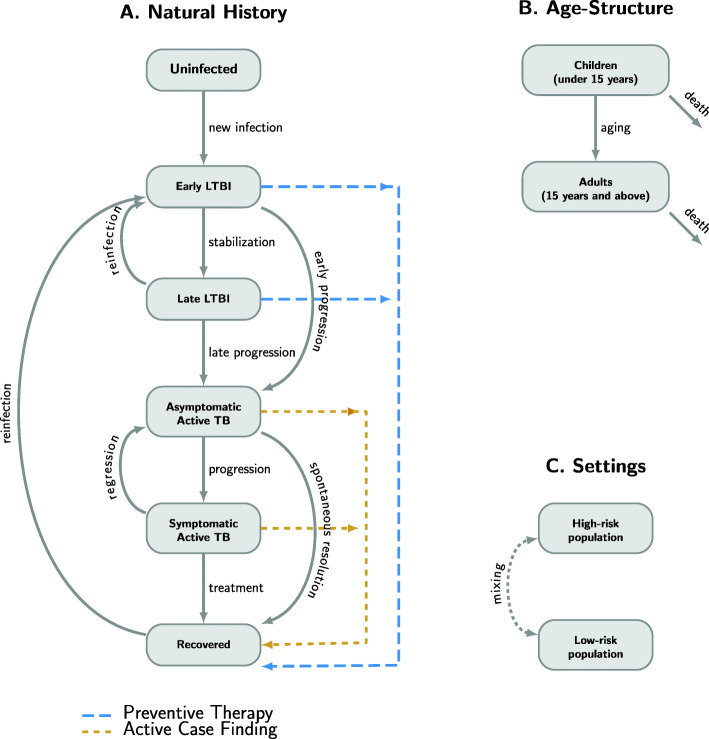
Schematic representation of the modeling approach. We use a compartmental modeling framework to incorporate **A** natural history of tuberculosis (TB), **B** age structure, and **C** risk groups. **A** Natural history was captured by modeling transition of individuals between six states: uninfected; two stages of latent TB infection (LTBI), early LTBI and late LTBI; two states of active TB disease, asymptomatic and symptomatic; and a recovered state. Uninfected individuals develop early LTBI upon acquiring TB infection, which can either stabilize to become late LTBI or progress early to active TB disease. Individuals with late LTBI can also develop active TB, through a late progression that occurs at a slower rate. Active TB is assumed to start in an asymptomatic form, which can either progress to a symptomatic form or resolve spontaneously to the recovered state without treatment. Symptomatic TB can either be diagnosed and treated or regress back to the asymptomatic form. Populations with late LTBI or who have recovered from previous TB disease can be reinfected (i.e., return to the early LTBI state) but are assumed to have partial immunity. Births and deaths, including TB-related deaths, are included in the model, but not shown here. Active case finding (followed by successful treatment) is modeled as a transition from the two active TB disease states to the recovered state; preventive therapy (and successful resolution of LTBI) is modeled as a transition from the LTBI states to recovered. **B** The population was subdivided into two groups based on age: children below 15 years and adults 15 years and above. Populations in the two age groups were modeled to have different TB prevalence (reflecting differences in natural history) and to be targeted differentially with the intervention. **C** The population was modeled to be living in either a high-risk area or other lower-risk areas of the city, with intermixing between the subpopulations, and with different TB transmission and diagnosis rates resulting in different TB prevalence in the two subpopulations

We developed a deterministic compartmental model of the natural history, transmission, and epidemiology of TB in a medium-sized city in India, represented schematically in Fig. 1. We modeled transitions between six TB-related states: an uninfected state, two states of LTBI (early LTBI and late LTBI), two states of active TB disease (asymptomatic and symptomatic), and a recovered state. In this conceptualization, individuals who acquire TB infection have a higher rate of developing active TB (early progression) during the first years following TB infection (early LTBI), followed by a lower rate (late progression) that persists unless LTBI is effectively treated. Active TB is assumed to start in an asymptomatic form that can either progress to symptomatic disease or resolve spontaneously without treatment. We assumed symptomatic TB is more infectious on a per-person-time basis and may result in death, cure through treatment, or spontaneous regression to asymptomatic disease. Individuals who have late LTBI or have recovered from previous TB disease can be reinfected but are assumed to have partial immunity from previous exposure.

To capture age-specific differences in TB natural history and intervention implementation, we divided the population into two age groups (< 15 and ≥ 15 years old). To capture heterogeneity in TB risk such as the greater risk associated with urban slums [15], we assumed that the city contains geographically distinct but intermixing subpopulations with higher and lower TB risk, whose differences in risk were modeled as differences in contact patterns (transmission rates) and in health care access (diagnosis rates and treatment success). For simplicity and interpretability, we assumed a closed population with no immigration or emigration. For model details, see Additional File 1:S-1 for model equations, and Additional File 1:Table S-1 for model parameters [16–25]. Model data and codes are available at the following repository: 10.5061/dryad.ttdz08kzg.

### Model calibration

**Fig. 2:**
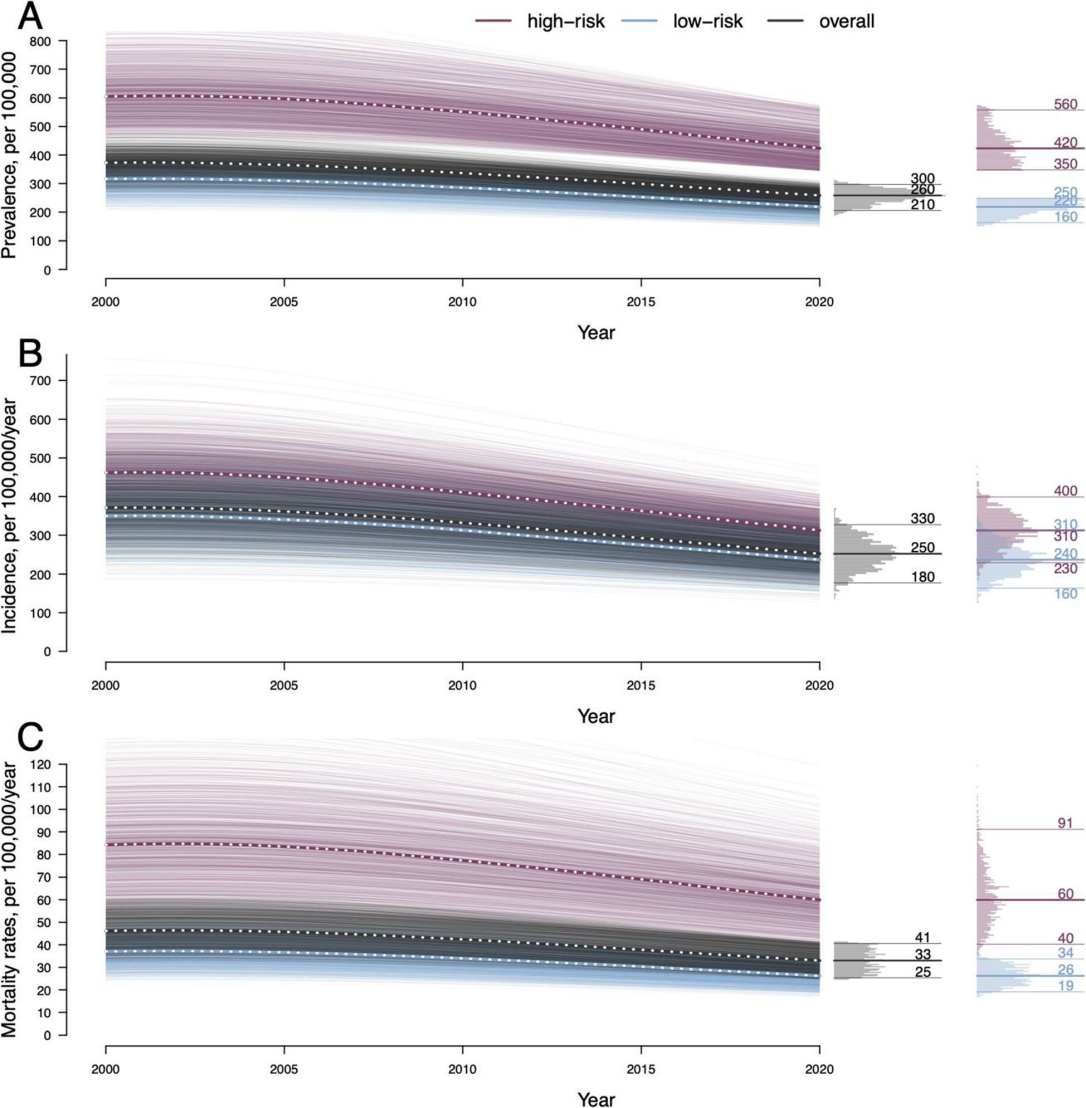
Simulations of the calibrated model. We calibrated the model to represent a mid-sized city in a high-burden country with TB epidemiology resembling that of India, with a population of 2 million in 2020; 24% were assumed to live in a high-prevalence area within the city, consistent with estimates of the slum-dwelling population [20]. Plotted curves represent simulations within the calibrated model. **A** The median prevalence of tuberculosis (TB) per 100,000 in 2020 was 260 (95% range 210–300) in the overall population, 420 (350–560) in the high-prevalence subpopulation, and 220 (160–250) in the low-risk subpopulation. **B** The median annual incidence of TB per 100,000 in 2020 was 250 (180–330) in the overall population and 310 (230–400) and 240 (160–310) in the higher- and lower-risk subpopulations, respectively. **C** The median annual TB-related mortality rate per 100,000 in 2020 was 33 (25–41) in the overall population and 60 (40–91) and 26 (19–34) in the high- and low-risk subpopulations, respectively. Additional model outputs and calibration targets are shown in Addtional file 1: Fig. S-1

We calibrated the model to key demographic and epidemiological features of TB in urban India: data-consistent calibration ranges for each target are listed in Table 1. The calibrated model comprised an equally weighted sample of simulations in which all calibrated model outputs were within their respective target ranges. The calibration process is described in detail in Additional File 1:S-2, key outputs of the calibrated model are shown in Fig. 2, and additional model outputs and their comparison with calibration targets are shown in Additional File 1:Fig. S-1.

**Table 1:** Model calibration targets and demographic assumptions

Target/assumption	Calibration range* or assumed value	Reference
Proportion of prevalent TB occurring among children under 15 years, in 2020	0.0975–0.1625	2018 WHO estimate for India of 0.13 [1]
Prevalence of TB in the low-risk subpopulation in 2020	150–250 per 100,000	Assumption of 200 per 100,000 in non-slum dwellers [15]
Prevalence of TB in the high-risk subpopulation in 2020	345–575 per 100,000	Assumption of 460 per 100,000 among slum-dwellers [26]
Annual rate of TB infection (ARTI) in the high-risk subpopulation in 2020	1.5–2.5%	Assumption of 2% mean rate [27]
Annual TB-related mortality rate in 2020	24.75–41.25 per 100,000	2018 WHO estimate for India of 33 per 100,00 [1]
Annual percentage decline in TB incidence, between 2000 and 2020	1.5–2.5%	WHO estimate for India between 2000 and 2018 of 2% [1]
Proportion of prevalent TB that is asymptomatic in 2020	0.45–0.75	Assumption of 0.6 mean proportion [28]
Proportion of urban population in the high-risk subpopulation	0.24	United Nation’s Millennium Development Goals database estimate of slum-dwelling population [29]
Proportion of the population under 15 years	0.27	Populationpyramid.net [30]
Annual birth rate	0.0197	Populationpyramid.net [30]

*The ranges are taken to be ± 25% around the point estimates from the corresponding references

### Interventions

**Fig. 3:**
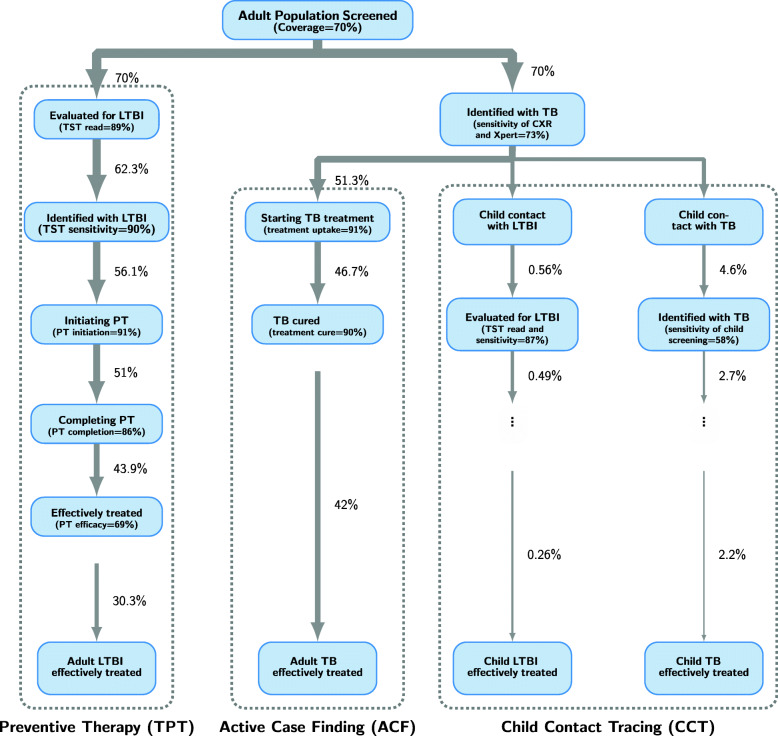
Flow chart illustrating the implementation of the one-time component of the intervention. Adults (15 years and above) are contacted at random as part of the one-time campaign; the intervention coverage (70% in the reference scenario) determines the proportion of adults who receive the initial steps of (i) screening with chest X-ray (CXR) for active tuberculosis (TB) (with Xpert Ultra testing if CXR suggests TB) and (ii) evaluation for latent TB (LTBI) using tuberculin skin test (TST). Those identified with LTBI (based on TST sensitivity) are offered preventive therapy; the proportion who successfully complete it (and move to the model’s recovered state) depends on the assumed levels of uptake, completion, and efficacy. Adults found to have active TB (dependent on the combined sensitivity of CXR and Xpert Ultra) are offered treatment; the proportion who move to the recovered state depends on the assumed uptake and treatment cure probability. For individuals identified with TB, all child contacts are further screened for both TB and LTBI (child contact tracing arm) and treated accordingly, similar to adults but accounting for the different sensitivity of TB testing in children and the small proportion of TB-affected children who are contacts of the adults diagnosed with active TB through the intervention. Baseline values for each parameter along the intervention cascade are included within parentheses, and the numbers next to the arrows indicate the cumulative percentage of prevalent cases among adults or children. Please refer to Additional file 1 Table S-2 in the supplementary materials for details on the derivation of these quantities

We conceptualized an intensive, city-wide intervention with two phases. The first phase (Fig. 3) comprised a one-time campaign to (a) screen the adult population, 15 years and above, for active TB (via a combination of chest X-ray and Xpert Ultra testing), with the treatment of persons identified with TB disease (active case finding, ACF) and evaluation of their child contacts, including treatment for active or latent TB (child contact tracing, CCT), and (b) test the same adult population (including adult household contacts) for latent TB, via tuberculin skin testing (TST), with the treatment of those with evidence of LTBI (preventive therapy, TPT). We assumed that the tuberculosis skin test (TST) would have 90% sensitivity in detecting (early or late) LTBI [31, 32] and that those with a positive TST result would be eligible for preventive therapy with 69% efficacy, consistent with the recently available 3HP regimen [33]. We modeled this campaign, including the two separate components, as occurring in an intensive but time-limited way in a single year (2020). At baseline, we assumed that 70% of the adult population would undergo screening for active TB. We incorporated a realistic cascade based on current literature for the sensitivity of active TB screening using mobile digital radiography; uptake and sensitivity of latent TB testing; uptake, completion, and effectiveness of TB treatment and preventive therapy; and the proportions of affected children who would be identifiable as contacts of a notified case (see Additional File 1:S-3 and Additional File 1:Table S-2 for additional details) [34–37].

The second phase of the intervention involved health system strengthening (HSS), modeled as a set of improvements to the TB health care system that could be facilitated by the improved infrastructure and lower TB burden achieved by the initial, one-time intervention phase, potentially allowing them to be implemented and maintained at lower incremental cost than if implemented on their own. Such systemic improvements could include (i) communication and outreach efforts to increase awareness of TB, improve access to TB services, and destigmatize TB; (ii) patient database supporting adherence support mechanisms such as telecall-based monitoring, patient welfare support during treatment, and digital adherence technology (DAT) to improve linkage to care and retention; (iii) retention of trained staff and infrastructural improvements, including in testing and screening equipment (achieved through investment in the one-time intervention), to improve quality of care and facilitate contact investigation; and (iv) enhanced surveillance and reporting with real-time analysis to enhance outbreak response and follow-up. The impact of these health system strengthening activities was modeled in the reference scenario as a 50% reduction in time to TB treatment initiation once symptomatic and a 50% reduction in treatment non-success (see Additional File 1:S-4 for additional details) [38–41].

### Model outcomes

The primary and secondary outcomes were respectively the total number of TB-related deaths and TB cases averted per million population over 10 years by the comprehensive two-phased campaign. To understand the contributions of each intervention component, we also evaluated the modeled intervention limited only to the one-time campaign (without subsequent HSS) and further evaluated an intervention of ACF and CCT alone (without TPT). We also modeled a “hypothetical TPT-only” comparison scenario, assuming the implementation of preventive therapy without any treatment of active TB. This scenario is artificial and hypothetical because in practice, any TPT intervention could not occur without first ruling out active TB (i.e., ACF). Nonetheless, its purpose in our current analysis is to serve as a comparator to disaggregate the relative impact of ACF and TPT. As a further comparator, we modeled a scenario with no intervention and current TB services continuing indefinitely. Outcomes were estimated by subtracting the number of projected TB cases and TB deaths in the intervention scenario from the projected numbers in the comparator scenario, and the reported uncertainty ranges represented uncertainties around natural history parameters.

### Sensitivity analyses

We evaluated the sensitivity of each epidemiological outcome to the value of each model parameter by comparing the subsets of model simulations that contained the top and bottom deciles of the corresponding parameter’s values among all calibrated simulations. We also evaluated the sensitivity of the same epidemiological outcomes to the degree of success in implementing each intervention component, by varying the population coverage (for the one-time intervention) and the magnitude of effect (for health system strengthening) (see Additional File 1:S-5 and Additional File 1:Figs S-2:S-6 for additional details).

## Results

In the calibrated model representing a medium-sized city in India, the median annual incidence of TB per 100,000 population prior to the intervention (i.e., at the start of 2020) was 250 (180–330) in the overall population: 310 (230–400) in the high-risk and 240 (160–310) in the low-risk subpopulation (Fig. 2). Between 2000 and 2020, the overall TB incidence was modeled as declining at 1.9% (1.5–2.5%) per year (Additional File 1:Fig. S-1). The median annual TB-related mortality rate per 100,000 in 2020 was 33 (25–41) in the overall population: 60 (40–91) and 26 (19–34) in the high- and low-risk subpopulations, respectively.

**Fig. 4:**
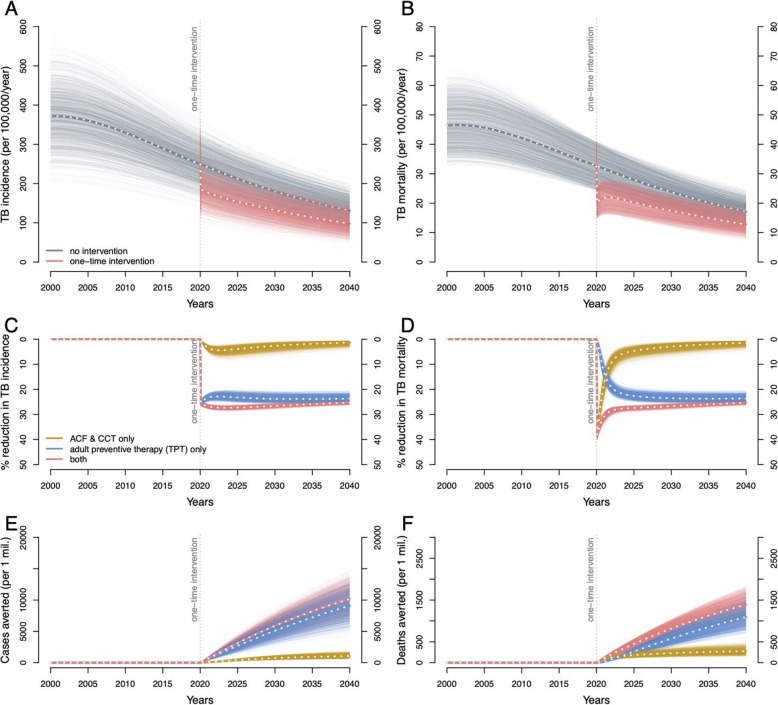
Projected epidemiological impact of a comprehensive one-time intervention to find and treat TB and LTBI. The projected TB incidence rate (**A**) and TB-related mortality rate (**B**), per 100,000 per year between 2000 and 2040, in model simulations without the intervention (gray) and in simulations with the intervention implemented in 2020 (red). Percentage reductions in annual TB incidence (**C**) and TB-related mortality (**D**) rates (red). Also shown are simulated interventions which (hypothetically) focused solely on treating adults for LTBI with no effect on active TB, represented by adult preventive therapy (PT) in Fig. 3 (blue), or focused solely on active case finding (ACF) and child contact tracing (CCT), represented by ACF and CCT arms in Fig. 3 (yellow). The cumulative impact is shown as cumulative TB cases averted (**E**) and cumulative TB-related deaths averted (**F**), for the full rapid one-time intervention (red) and for its PT (blue) and ACF/CCT (yellow) components separately

We projected that, after accounting for imperfect test sensitivity, losses to follow-up, and imperfect completion of treatment (see supplementary materials for details), one-time screening of 70% of the adult population would result in the successful treatment of 42% of adults with prevalent active TB (Fig. 3, ACF) and 30% of adults with LTBI (Fig. 3, TPT). By screening and treating child contacts for both active TB and LTBI, an additional 2.2% of children (under 15) with active TB and 0.26% of children with LTBI could be successfully treated (Fig. 3, CCT). Even in the absence of lasting effects on care delivery by the health system, this one-time intervention was projected to result in a 26.6% (95% range 25.7–27.4%) reduction in city-wide annual TB incidence (Fig. 4A, C, red lines) and a 26.8% (25.9–27.6%) reduction in annual TB mortality (Fig. 4B, D, red lines), at 10 years following implementation. This impact was achieved immediately—with an estimated 26.7% (25.9–27.5%) reduction in TB incidence and 31.5% (30.0–33.4%) reduction in TB mortality within 1 year—and persisted over time, lowering the TB incidence by 25.3% (24.1–26%) and TB mortality by 25.3% (24.2–26%) over the next 20 years following the intervention. Consequently, the cumulative impact of the one-time campaign was substantial and sustained: per 1 million population, a projected 5840 (4060–7650) cases would be averted by year 10 and 10,100 (6930–13,500) cases by year 20 (Fig. 4E, red lines); corresponding lives saved were 809 (612–1010) by year 10 and 1380 (1020–1750) by year 20 (Fig. 4F, red lines).

If this one-time campaign was limited to only active case finding and related components, it achieved an immediate impact, but one that was not sustained: y curing 42% of people with prevalent active TB, this case-finding-only intervention was projected to avert 81 (61–103) deaths (Fig. 4F, yellow lines) per million population averted in year 1, but only 149 (95–227) cumulatively in years 2 through 10. By contrast, when hypothetically limited to LTBI diagnosis and treatment (i.e., ignoring any effect on active TB), the immediate effects were small, but the longer-term impact was more pronounced: only 18 (11–26) deaths (Fig. 4F, blue lines) were averted by year 1, but an additional 553 (409–716) deaths were averted in years 2 through 10 (Fig. 6B, C). Consequently, by year 10, the cumulative impact of the combined campaign predominantly reflected the impact of LTBI treatment: 71% of the averted deaths and 87% of the averted cases were achieved through treatment of LTBI. However, on a per-person-treated basis, successfully treating an individual with prevalent active TB was still 10–30 times more impactful in averting future TB cases, compared to successfully treating an individual with LTBI (Additional File 1:Fig. S-3).

**Fig. 5:**
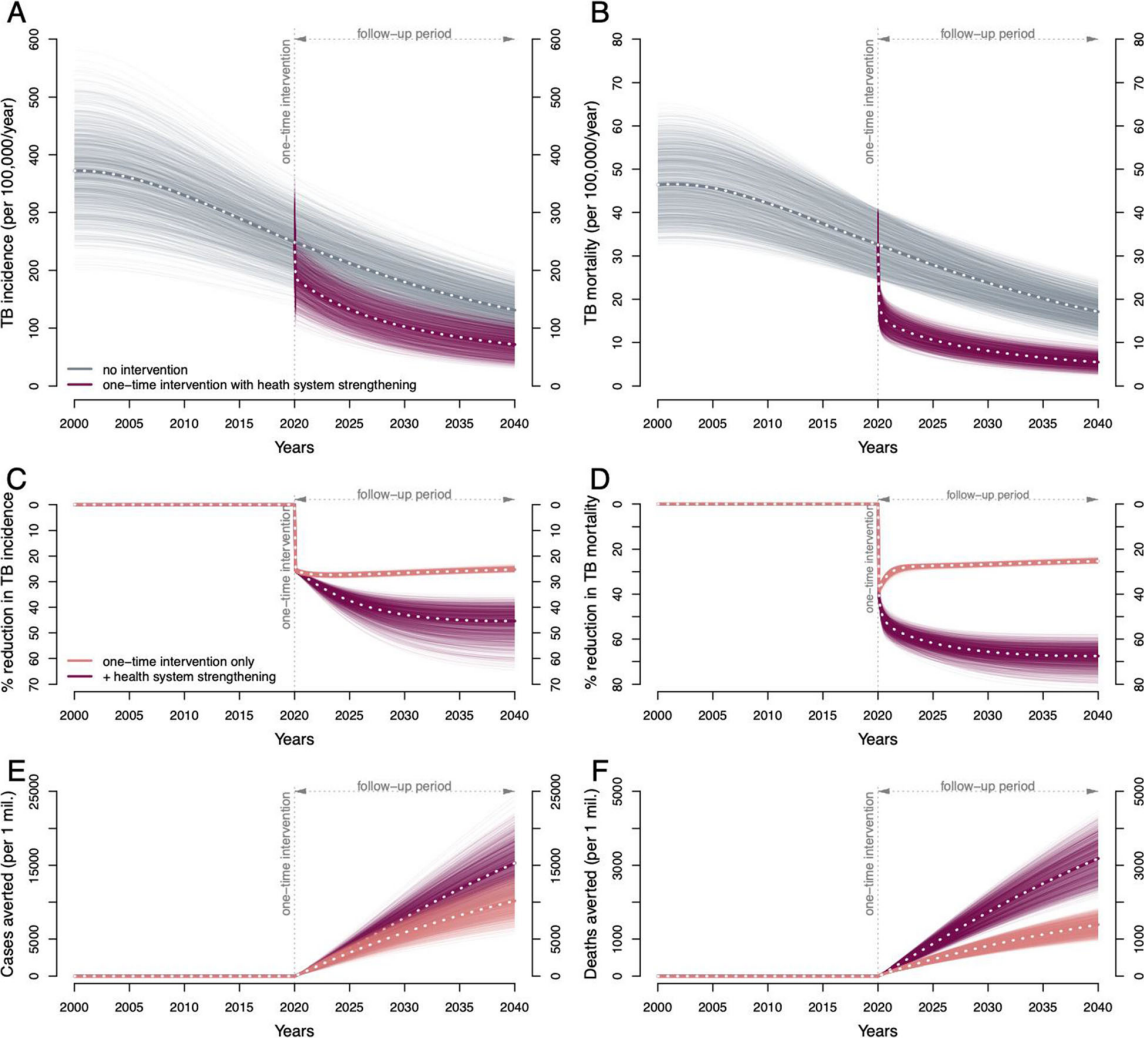
The impact of a comprehensive one-time intervention supplemented by follow-up health system strengthening. The TB incidence rate (**A**) and TB-related mortality (**B**) rate, per 100,000 per year between 2000 and 2040, in model simulations without the intervention (gray), and with a one-time intervention implemented in 2020, including medium-term health system strengthening after 2020 (violet). Percentage reductions in TB incidence (**C**) and TB-related mortality (**D**) rates (darker burgundy). Also shown for comparison are reductions with the one-time intervention only (lighter red). Cumulative cases of TB (**E**) and cumulative TB-related deaths (**F**) averted per 1 million by the full intervention (burgundy); the cumulative impacts of the one-time intervention alone are also shown for comparison (red)

The inclusion of follow-up activities to strengthen TB health systems substantially augmented the impact of the intervention. Annual TB incidence could be reduced by 42.9% (37–52.3%) and mortality by 65.6% (59.3–73.4%) at 10 years compared to no intervention (Fig. 5C, D, dark burgundy lines). Consequently, if the one-time campaign could catalyze health system strengthening, the total number of cases and deaths averted by year 10 increased to 7800 (5450–10,200; a 1.3-fold increase compared to the one-time intervention only) and 1710 (1290–2180; a 2.1-fold increase), respectively, per million population.

**Fig. 6:**
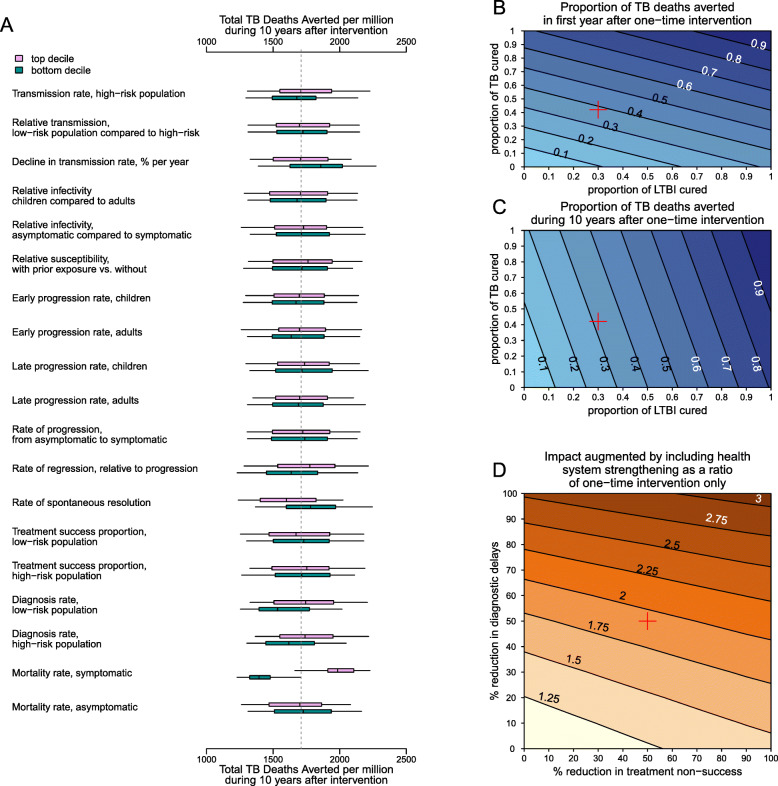
Sensitivity analyses. **A** Sensitivity of the primary outcome (TB-related deaths averted over 10 years by a combined intervention of a one-time campaign plus health system strengthening), to individual model parameters. Each pair of boxplots shows variation in the outcome when the analysis was limited to either simulations in which the value of the parameter of interest was in the top (light pink) or bottom (dark green) decile of its values across all accepted simulations. In each boxplot, the edges of the box represent the lower and upper interquartile range, the band in the middle represents the median, and the end of the whiskers represent 2.5th and 97.5th percentiles. The vertical dotted line shows the median across all accepted simulations. **B**, **C** Contours show the proportion of TB deaths averted by year 1 (**B**) and by year 10 (**C**) after a one-time campaign (with no subsequent health system strengthening) that achieves cure of LTBI in the proportion of the population indicated on the *x*-axis and cure of active TB in the proportion indicated on the *y*-axis. **D** Colored level surfaces indicate the additional impact on mortality of including health system strengthening measures with a one-time campaign, relative to the impact of the one-time campaign alone, assuming 70% coverage with one-time intervention, and a specified percentage reduction in unsuccessful treatments (*x*-axis) and diagnostic delays (*y*-axis). The red cross in **B**–**D** indicates the reference scenario. Please see Additional file 1: Fig. S-2 for the sensitivity of the secondary outcome, TB cases averted over 10 years

Both primary and secondary outcomes were robust to uncertainties around most model parameters. When comparing simulations containing the highest 10% versus lowest 10% values of any model parameter, the most influential parameter for the outcome of deaths averted was the TB-related mortality rate among individuals with symptomatic TB (resulting in < 20% change in median projection, compared to the primary estimate) (Fig. 6A), and the most influential parameters for TB cases averted were the rates of early TB progression and spontaneous resolution (< 30% change, Additional File 1:Fig S2). The projected impact of the intervention varied in proportion to intervention magnitude (Fig. 6B, C). The impact of health system strengthening likewise reflected the achievable magnitude of reductions in both treatment delays and treatment non-success (Fig. 6D).

## Discussion

This modeling analysis suggests that an intensive, short-term intervention can feasibly achieve rapid and sustained reductions in TB incidence and mortality if it is (i) implemented in a community-wide fashion reaching a large proportion of the target population, (ii) comprehensive in treating both TB disease and LTBI, and (iii) able to leverage infrastructure to catalyze medium-term health system strengthening. Specifically, we estimated that a realistic intervention in an urban Indian setting could reduce TB mortality by nearly two-thirds over 10 years, saving 1710 lives per million population in the process. Considering that TB kills more people than any other infectious agent (except SARS-CoV-2 during the pandemic), both in India and globally, the number of lives saved would likely rival that of almost any other intervention to fight an infectious disease.

These results underscore that, while case-finding and treatment of active TB are critical for achieving rapid reductions in TB burden, coupling this with the treatment of LTBI and health system strengthening is essential for longer-term impact. In this analysis, more than 70% of TB deaths and up to 87% of TB cases averted over a 10-year period after a one-time intervention were attributable to treating LTBI. These findings are consistent with previous research highlighting the importance of preventive therapy in achieving long-term success against TB [42]. Furthermore, the achievable 10-year reduction in TB deaths more than doubled when the one-time intervention was coupled with health system strengthening that improved care delivery over the medium term. These results should not, however, diminish the importance of finding and treating active TB in the short term. Not only is ruling out active TB essential for LTBI treatment but the number of (downstream) cases averted by treatment of a prevalent active TB is 10–30 times greater than those averted by treating one person with LTBI (Additional file 1:Fig. S-3). Nevertheless, since the prevalence of LTBI is nearly 100 times larger than the prevalence of TB disease (median prevalence of LTBI 39% compared to the median prevalence of TB of 420 per 100,000, Additional file 1:Fig. S-1), treatment of LTBI plays a major role in reducing TB burden.

Since treatment of LTBI is critical to achieving large reductions in incidence and mortality, barriers to achieving high coverage of LTBI treatment also limit the overall effectiveness of large-scale TB interventions. Accounting for existing gaps in the LTBI care cascade—as done here—is important to inform realistic expectations for what an intensive one-time intervention can achieve. Previous studies have highlighted substantial gaps in the cascade of LTBI care and challenges in the implementation of LTBI treatment [43]. Thus, not only does a high-impact TB prevention campaign require accurate LTBI diagnostic tests and high-efficacy preventive regimens, but it also requires strategies to achieve high uptake and completion of LTBI treatment. Shorter treatment regimens can improve completion [44]; maximizing the impact of such regimens will require combining them with additional innovative approaches to help augment the cascade of LTBI diagnosis and treatment [45]. The use of tests for LTBI with imperfect specificity (e.g., TST) may result in overdiagnosis and treatment of people without underlying LTBI, which are not captured in the epidemiological impact studied here, but will have consequences for resource use and costs.

The high prevalence of TB in many high-burden settings also reflects underlying weaknesses in health care systems [46, 47]. These weaknesses often manifest as limited access to care, prolonged delays in diagnosis and treatment [39], losses to follow-up, and treatment non-success [41]. Our results underscore the potential impact of HSS and also highlight the opportunity of leveraging a one-time campaign to mitigate some of these underlying weaknesses in the TB health care system—though strengthening health systems will have an important impact on TB, even in the absence of a one-time biomedically focused intervention as modeled here. Specifically, we found that HSS could more than double the number of lives saved by a comprehensive, one-time, TB-focused intervention. Even a modest 20% reduction in diagnostic delay alone augmented this 10-year impact by 25% (Fig. 6D). Hence, in designing major interventions to find and treat people with TB (and LTBI), implementers must recognize the importance of sustained improvement of TB care over the longer term—potentially by leveraging those short-term activities without incurring substantial additional costs.

As an example of the potential for large-scale interventions to be economically viable, reduced TB caseloads achieved by a one-time intervention could free up resources to enhance control efforts or maintain quality of care. Data collected as part of the initial intervention could be used to identify high-risk populations and/or gaps in the existing system of TB care that could be closed with targeted interventions. Furthermore, the investment that would be made during the initial intervention to reach, test, and treat a substantial proportion of the community could be leveraged to increase general awareness of TB and TB services in the population improve patient detection and adherence (e.g., by building comprehensive patient/population database) and improve quality of patient care (e.g., by retaining equipment and staffs trained for the initial intervention). HSS has the potential to be impactful in high-incidence settings like India, whether combined with a larger-scale TB-focused biomedical intervention (as modeled here) or as a stand-alone approach. As such, HSS should be considered a critical component of any coordinated TB response, regardless of whether biomedical TB interventions are also being planned. That HSS measures may be further enabled by an intensive campaign to find and treat both active and latent TB lends further weight to the argument for adopting such a combined approach. As such, high priority should be assigned to implementation research to identify specific programs that improve the TB care cascade in specific settings (especially as performed after major case-finding and treatment campaigns) and to assess the generalizability of such programs across settings.

These findings are subject to certain limitations. The impact of these interventions could be higher if targeted to specific high-risk populations that bear a larger burden of TB and contribute disproportionately to transmission. However, identifying such populations and achieving high coverage therein may present its own challenges. We included a high-risk population in our model to capture some of this heterogeneity, but we found that at high levels of coverage in the overall population, targeting the intervention preferentially to this high-risk population did not substantially increase the overall impact of the intervention (Additional File 1:Fig. S-4). Furthermore, the added value of targeting interventions to such high-risk populations depends on the factors that are setting-specific and difficult to precisely measure, such as the variation in TB prevalence over small geographic scales and the degree of mixing and transmission between subpopulations [48]. With early trial results suggesting that novel TB vaccines are moderately efficacious in reducing the risk of TB disease [49], future modeling work could also consider incorporating vaccination as a part of a comprehensive intervention. The durability and efficacy of vaccine-derived protection are likely to be critical considerations for such analyses. For simplicity, we conceptualized a closed population with no immigration or emigration. The actual impact of an intervention, when evaluated only in the population where the intervention was conducted, is likely to be diminished by such migration. However, when migration occurs, the intervention’s effect on emigrants’ risk of TB will extend the benefits of the intervention beyond the local population. Finally, we adopted a number of simplifying assumptions and data choices to broadly represent both an urban population-center in India and implementation of a comprehensive mass intervention; the projections created here are therefore not fully reflective of the impact of any specific intervention as implemented in a specific (and inherently more complex) epidemiological setting.

We made several modeling choices to represent the natural history of TB, in which data are either sparse or open to multiple interpretations. (i) We assumed that TB infection imparts partial immunity to reinfection (because individuals with evidence of LTBI are less likely to develop TB disease after reexposure [18, 19]) and that this immune protection extends to those who were previously treated (for both LTBI and TB disease) or those who spontaneously resolved. The inclusion of protection for those who had received treatment is consistent with other TB modeling literature [50, 51], and has little impact on our main results, but it means that our model relies solely on relapse and geographic risk heterogeneity to account for the higher risk of recurrence faced after treatment for TB disease [52]. (ii) Relatedly, we modeled the rate of late progression of LTBI to be non-zero; recent analyses have argued that remote infections rarely progress after a few years [53] or may even become sterilized without antitubercular therapy [54]. The true risk of progression among individuals with remote TB infection remains poorly understood [23] but can be consequential for the impact of these interventions. In our simulations, we found that the impact in terms of cases averted was about 30% larger in simulations where the rates of late progression were in the top decile, compared to the bottom decile (Additional File 1:Fig. S-2). In addition, if preventive therapy could be targeted to individuals with recent infection (e.g., if diagnostic assays for recent infection could be developed), up to 89% of cumulative 10-year TB mortality could be averted, while only delivering preventive therapy to one-tenth of the infected population (Additional File 1:S-6). Furthermore, if the reactivation rates of remote TB reactivation (and consequently the lifetime benefits of TPT) are substantially lower, it will be important to carefully weigh the risks and benefits of TPT. Prioritizing populations based on risks, such as household contacts of known TB cases and those with underlying risk factors, may be necessary. (iii) Finally, our model included the potential for TB disease to resolve without treatment (via regression and spontaneous resolution); the estimated rates of regression and resolution varied over a wide range in our calibrated models, reflecting not just uncertainties in these estimates but also a correlation with other parameters (e.g., late progression rates). Rates of resolution in the bottom decile, which were closer to estimates in a recent study [24], yielded estimates of cases averted that were up to 15% smaller than the median estimate (Additional File 1:Fig. S-2). However, the estimated number of deaths averted was more robust to these parameter choices (Fig. 6).

In this analysis, we focused on the epidemiological outcomes and did not consider estimates of costs. A natural next step would be to assess the costs of implementing a comprehensive one-time intervention of this scale and to estimate the potential benefits in terms of future costs that could be saved, as well as mortality and morbidity that could be averted. Furthermore, a comprehensive cost-effectiveness analysis of our proposed two-phased approach would also need to evaluate the feasibility and costs of incorporating health system strengthening activities into large case-finding campaigns.

## Conclusions

In conclusion, this modeling study suggests that a focused and intensive intervention to halt TB transmission in a high-burden setting, leveraged to also strengthen subsequent TB care by the routine health system, can reduce TB incidence by over 40% (7800 cases averted per million population) and TB mortality by almost two-thirds (1710 lives saved per million population) over a 10-year period. Such impact would represent a substantial acceleration of the currently modest decline in TB burden seen throughout the world in recent years. These impacts can be achieved rapidly—with much of the reductions occurring in the first year—and can be sustained for decades. The success of such an intervention, however, is closely tied to the ability to effectively treat LTBI and to strengthen the TB cascade of care through an initial investment. A rapid and sustained “step change” in TB burden is therefore achievable, but only with a comprehensive approach that includes case-finding and treatment of active TB, treatment of LTBI, and long-term strengthening of the TB health care system.

## Supplementary Information

**Additional file 1:.** S-1. Model details. S-2. Model calibration. S-3. Modeling the effects of the one-time intervention. S-4. Modeling the effects of medium-term health system strengthening. S-5. Sensitivity analyses. Figure S-1. Comparison of model simulations, and calibration targets. Figure S-2. Sensitivity analyses of the secondary outcome, TB cases averted over 10 years by a combined intervention of a one-time campaign plus health system strengthening. Figure S-3. Comparing the impact of curing LTBI versus TB disease. Figure S-4. The impact of a one-time intervention (without health system strengthening) when the intervention was targeted to the high-risk population. Figure S-5. The impact of a one-time intervention (without health system strengthening), with shorter duration of early LTBI. Figure S-6. The impact of the full intervention when preventive therapy is limited to recent infections. Table S-1. Model parameters. Table S-2. Model parameters for one-time intervention.

## Data Availability

Data and model codes can be accessed at the following repository: 10.5061/dryad.ttdz08kzg.

## Abbreviations

ACF: Active case finding
CCT: Child contact tracing
CXR: Chest X-ray
HIV: Human immunodeficiency virus
HSS: Health system strengthening
LTBI: Latent tuberculosis infection
TB: Tuberculosis
TPT: Tuberculosis preventive therapy
TST: Tuberculin skin test

## References

[1] World Health Organization. Global Tuberculosis Report. Geneva. Switzerland. 2020;2020.

[2] World Health Organization END TB strategy, 2015. Available at: http://www.who.int/tb/post2015_strategy/en/.

[3] Comstock GW, Philip RN (1961). Decline of the tuberculosis epidemic in Alaska. Public Health Rep..

[4] Marks GB, Nguyen NV, Nguyen PT, Nguyen TA, Nguyen HB, Tran KH, Nguyen SV, Luu KB, Tran DT, Vo QT, Le OT (2019). Community-wide screening for tuberculosis in a high-prevalence setting. New England Journal of Medicine..

[5] Paolo WF, Nosanchuk JD (2004). Tuberculosis in New York City: recent lessons and a look ahead. The Lancet infectious diseases..

[6] Go U, Park M, Kim UN, Lee S, Han S, Lee J, Yang J, Kim J, Park S, Kim Y, Yoo H (2018). Tuberculosis prevention and care in Korea: evolution of policy and practice. Journal of Clinical Tuberculosis and Other Mycobacterial Diseases..

[7] Qin ZZ, Sander MS, Rai B, Titahong CN, Sudrungrot S, Laah SN, Adhikari LM, Carter EJ, Puri L, Codlin AJ, Creswell J. Using artificial intelligence to read chest radiographs for tuberculosis detection: a multi-site evaluation of the diagnostic accuracy of three deep learning systems. Scientific Reports. 2019;9(1):1-0. 10.1038/s41598-019-51503-3 PMC6802077 31628424

[8] Sterling TR, Villarino ME, Borisov AS, Shang N, Gordin F, Bliven-Sizemore E, Hackman J, Hamilton CD, Menzies D, Kerrigan A, Weis SE (2011). Three months of rifapentine and isoniazid for latent tuberculosis infection. New England Journal of Medicine..

[9] Dorman SE, Schumacher SG, Alland D, Nabeta P, Armstrong DT, King B, Hall SL, Chakravorty S, Cirillo DM, Tukvadze N, Bablishvili N (2018). Xpert MTB/RIF Ultra for detection of Mycobacterium tuberculosis and rifampicin resistance: a prospective multicentre diagnostic accuracy study. The Lancet Infectious Diseases..

[10] Menzies NA, Cohen T, Lin HH, Murray M, Salomon JA (2012). Population health impact and cost-effectiveness of tuberculosis diagnosis with Xpert MTB/RIF: a dynamic simulation and economic evaluation. PLoS Med..

[11] Cohen T, Lipsitch M, Walensky RP, Murray M (2006). Beneficial and perverse effects of isoniazid preventive therapy for latent tuberculosis infection in HIV–tuberculosis coinfected populations. Proceedings of the National Academy of Sciences..

[12] Kasaie P, Andrews JR, Kelton WD, Dowdy DW (2014). Timing of tuberculosis transmission and the impact of household contact tracing. An agent-based simulation model. American Journal of Respiratory and Critical Care Medicine..

[13] Hill PC, Dye C, Viney K, Tabutoa K, Kienene T, Bissell K, Williams BG, Zachariah R, Marais BJ, Harries AD (2014). Mass treatment to eliminate tuberculosis from an island population. The International Journal of Tuberculosis and Lung Disease..

[14] Houben RM, Menzies NA, Sumner T, Huynh GH, Arinaminpathy N, Goldhaber-Fiebert JD (2016). Feasibility of achieving the 2025 WHO global tuberculosis targets in South Africa, China, and India: a combined analysis of 11 mathematical models. Lancet Glob Health..

[15] Pandey S, Chadha VK, Laxminarayan R, Arinaminpathy N (2017). Estimating tuberculosis incidence from primary survey data: a mathematical modeling approach. The International Journal of Tuberculosis and Lung Disease..

[16] Dye C, Garnett GP, Sleeman K, Williams BG (1998). Prospects for worldwide tuberculosis control under the WHO DOTS strategy. The Lancet..

[17] Basu S, Orenstein E, Galvani AP (2008). The theoretical influence of immunity between strain groups on the progression of drug-resistant tuberculosis epidemics. The Journal of Infectious Diseases..

[18] Andrews JR, Noubary F, Walensky RP, Cerda R, Losina E, Horsburgh CR (2012). Risk of progression to active tuberculosis following reinfection with Mycobacterium tuberculosis. Clinical Infectious Diseases..

[19] Vynnycky E, Fine PE (1997). The natural history of tuberculosis: the implications of age-dependent risks of disease and the role of reinfection. Epidemiology & Infection..

[20] Shrestha S, Chatterjee S, Rao KD, Dowdy DW (2016). Potential impact of spatially targeted adult tuberculosis vaccine in Gujarat, India. Journal of The Royal Society Interface..

[21] Vynnycky E, Fine PE (1997). The annual risk of infection with Mycobacterium tuberculosis in England and Wales since 1901. The International Journal of Tuberculosis and Lung Disease..

[22] Ragonnet R, Trauer JM, Scott N, Meehan MT, Denholm JT, McBryde ES (2017). Optimally capturing latency dynamics in models of tuberculosis transmission. Epidemics..

[23] Dale KD, Karmakar M, Snow KJ, Menzies D, Trauer JM, Denholm JT. Quantifying the rates of late reactivation tuberculosis: a systematic review. The Lancet Infectious Diseases. 2021; In press. 10.1016/S1473-3099(20)30728-3. 10.1016/S1473-3099(20)30728-3 33891908

[24] Ragonnet R, Flegg JA, Brilleman SL, Tiemersma EW, Melsew YA, McBryde ES, Trauer JM. Revisiting the natural history of pulmonary tuberculosis: a Bayesian estimation of natural recovery and mortality rates. Clinical Infectious Diseases. 2020:ciaa602. 10.1093/cid/ciaa602 32766718

[25] Kendall EA, Shrestha S, Cohen T, Nuermberger E, Dooley KE, Gonzalez-Angulo L, Churchyard GJ, Nahid P, Rich ML, Bansbach C, Forissier T (2017). Priority-setting for novel drug regimens to treat tuberculosis: an epidemiologic model. PLoS Medicine..

[26] Dhanaraj B, Papanna MK, Adinarayanan S, Vedachalam C, Sundaram V, Shanmugam S, Sekar G, Menon PA, Wares F, Swaminathan S (2015). Prevalence and risk factors for adult pulmonary tuberculosis in a metropolitan city of South India. PloS One..

[27] Gopi PG, Venkatesh Prasad V, Vasantha M, Subramani R, Tholkappian AS, Sargunan D, Narayanan PR (2008). Annual risk of tuberculosis infection in Chennai city. Indian Journal of Tuberculosis..

[28] Onozaki I, Law I, Sismanidis C, Zignol M, Glaziou P, Floyd K (2015). National tuberculosis prevalence surveys in Asia, 1990–2012: an overview of results and lessons learned. Tropical Medicine & International Health..

[29] United Nations. Millennium Development Goals. Accessed at http://mdgs.un.org/unsd/mdg/Data.aspx?cr=356 (July 12, 2020).

[30] Population Pyramids of the World from 1950 to 2100. Accessed at https://www.populationpyramid.net/india/2019/ (July 12, 2020)

[31] Kik SV, Franken WP, Mensen M, Cobelens FG, Kamphorst M, Arend SM, Erkens C, Gebhard A, Borgdorff MW, Verver S (2010). Predictive value for progression to tuberculosis by IGRA and TST in immigrant contacts. European Respiratory Journal..

[32] Dick Menzies, 2020. Use of the tuberculin skin test for diagnosis of latent tuberculosis infection (tuberculosis screening) in adults - UpToDate. Accessed at https://www.uptodate.com/contents/use-of-the-tuberculin-skin-test-for-diagnosis-of-latent-tuberculosis-infection-tuberculosis-screening-in-adults on Nov 19, 2020.

[33] Pease C, Hutton B, Yazdi F, Wolfe D, Hamel C, Quach P, Skidmore B, Moher D, Alvarez GG (2017). Efficacy and completion rates of rifapentine and isoniazid (3HP) compared to other treatment regimens for latent tuberculosis infection: a systematic review with network meta-analyses. BMC Infectious Diseases..

[34] World Health Organization. Chest radiography in tuberculosis detection; summary of current WHO recommendations and guidance on programmatic approaches. Accessed at https://www.who.int/tb/publications/chest-radiography/en/ on Jan 4, 2021.

[35] Atherton RR, Cresswell FV, Ellis J, Kitaka SB, Boulware DR (2019). Xpert MTB/RIF Ultra for tuberculosis testing in children: a mini-review and commentary. Frontiers in Pediatrics..

[36] WHO Tuberculosis Research Office (1955). FURTHER studies of geographic variation in naturally acquired tuberculin sensitivity. Bulletin of the World Health Organization.

[37] Borgdorff MW, Van Soolingen D (2013). The re-emergence of tuberculosis: what have we learnt from molecular epidemiology?. Clinical Microbiology and Infection..

[38] Mundra A, Kothekar P, Deshmukh PR, Dongre A (2019). Why tuberculosis patients under revised national tuberculosis control programme delay in health-care seeking? A mixed-methods research from Wardha District. Maharashtra. Indian Journal of Public Health..

[39] Bronner Murrison L, Ananthakrishnan R, Swaminathan A, Auguesteen S, Krishnan N, Pai M, Dowdy DW (2016). How do patients access the private sector in Chennai, India? An evaluation of delays in tuberculosis diagnosis. The International Journal of Tuberculosis and Lung Disease.

[40] Mistry N, Rangan S, Dholakia Y, Lobo E, Shah S, Patil A (2018). Durations and delays in care seeking, diagnosis and treatment initiation in uncomplicated pulmonary tuberculosis patients in Mumbai. India. PloS One..

[41] Subbaraman R, Nathavitharana RR, Satyanarayana S, Pai M, Thomas BE, Chadha VK, Rade K, Swaminathan S, Mayer KH (2016). The tuberculosis cascade of care in India’s public sector: a systematic review and meta-analysis. PLoS Medicine..

[42] Dye C, Glaziou P, Floyd K, Raviglione M (2013). Prospects for tuberculosis elimination. Annual Review of Public Health..

[43] Alsdurf H, Hill PC, Matteelli A, Getahun H, Menzies D (2016). The cascade of care in diagnosis and treatment of latent tuberculosis infection: a systematic review and meta-analysis. The Lancet Infectious Diseases..

[44] Menzies D, Adjobimey M, Ruslami R, Trajman A, Sow O, Kim H, Obeng Baah J, Marks GB, Long R, Hoeppner V, Elwood K (2018). Four months of rifampin or nine months of isoniazid for latent tuberculosis in adults. New England Journal of Medicine..

[45] Fox GJ, Dobler CC, Marais BJ, Denholm JT (2017). Preventive therapy for latent tuberculosis infection—the promise and the challenges. International Journal of Infectious Diseases..

[46] Atun R, Weil DE, Eang MT, Mwakyusa D (2010). Health-system strengthening and tuberculosis control. The Lancet..

[47] Creswell J, Codlin AJ, Andre E, Micek MA, Bedru A, Carter EJ, Yadav RP, Mosneaga A, Rai B, Banu S, Brouwer M (2014). Results from early programmatic implementation of Xpert MTB/RIF testing in nine countries. BMC Infectious Diseases..

[48] Dowdy DW, Golub JE, Chaisson RE, Saraceni V (2012). Heterogeneity in tuberculosis transmission and the role of geographic hotspots in propagating epidemics. Proceedings of the National Academy of Sciences..

[49] Tait DR, Hatherill M, Van Der Meeren O, Ginsberg AM, Van Brakel E, Salaun B, Scriba TJ, Akite EJ, Ayles HM, Bollaerts A, Demoitié MA (2019). Final analysis of a trial of M72/AS01E vaccine to prevent tuberculosis. New England Journal of Medicine..

[50] Houben RM, Dodd PJ (2016). The global burden of latent tuberculosis infection: a re-estimation using mathematical modelling. PLoS Medicine..

[51] Menzies NA, Cohen T, Hill AN, Yaesoubi R, Galer K, Wolf E, Marks SM, Salomon JA (2018). Prospects for tuberculosis elimination in the United States: results of a transmission dynamic model. American Journal of Epidemiology..

[52] Marx F, Yaesoubi R, Menzies NA, Salomon JA, Bilinski A, Beyers N, Cohen T (2018). Tuberculosis control interventions targeted to previously treated people in a high incidence setting: a modelling study. The Lancet Global Health..

[53] Behr MA, Edelstein PH, Ramakrishnan L. Revisiting the timetable of tuberculosis. BMJ. 2018;362. 10.1136/bmj.k2738 PMC6105930 30139910

[54] Emery JC, Richards AS, Dale KD, McQuaid CF, White RG, Denholm JT, Houben RMGJ (2021). Self-clearance of mycobacterium tuberculosis infection: implications for lifetime risk and population at-risk of tuberculosis disease. Proceedings of the Royal Society B..

