# Supplementary material for: Achieving a “step change” in the tuberculosis epidemic through comprehensive community-wide intervention: a model-based analysis
Source: BMC Med. 2021 Oct 14;19:244. doi: 10.1186/s12916-021-02110-5 (PMC8514283; doi:10.1186/s12916-021-02110-5)
Supplement: Supplementary file 1 — Additional file 1:. S-1. Model details. S-2. Model calibration. S-3. Modeling the effects of the one-time intervention. S-4. Modeling the effects of medium-term health system strengthening. S-5. Sensitivity analyses. Figure S-1. Comparison of model simulations, and calibration targets. Figure S-2. Sensitivity analyses of the secondary outcome, TB cases averted over 10 years by a combined intervention of a one-time campaign plus health system strengthening. Figure S-3. Comparing the impact of curing LTBI versus TB disease. Figure S-4. The impact of a one-time intervention (without health system strengthening) when the intervention was targeted to the high-risk population. Figure S-5. The impact of a one-time intervention (without health system strengthening), with shorter duration of early LTBI. Figure S-6. The impact of the full intervention when preventive therapy is limited to recent infections. Table S-1. Model parameters. Table S-2. Model parameters for one-time intervention. [file 12916_2021_2110_MOESM1_ESM.pdf]

Additional File 1
Supplementary Materials for: Achieving a "step change" in the tuberculosis epidemic through comprehensive community-wide intervention: a model-based analysis.

Sourya Shrestha[1,*], Emily Kendall[2], Rebekah Chang[3], Roy Joseph[3], Laura Gillini[3], Parastu Kasaie[1], Anthony Todd Fojo[2], Michael Campbell[3], Nimalan Arinamipathy[4], David W. Dowdy[1]

[1]Department of Epidemiology, Johns Hopkins School of Public Health, Baltimore, USA

[2]Johns Hopkins School of Medicine, Baltimore, USA

[3]Clinton Health Access Initiative, Boston, USA

[4]Imperial College, London, UK

[*]To whom correspondence should be addressed; E-mail: sourya@jhu.edu.

S-1 Model details

The model we developed evaluates the impact of a comprehensive one-time intervention and follow-up health system strengthening. It was structured to take into account natural history and transmission of TB, including buden of subclinical TB and future progression of LTBI, and aging of the population and screening of the population based on age. The model is described and schematically presented in the main text. Here, we provide the mathematical expressions of the ordinary differential equations that describe the model in the entirety. Let $X_{\{i,j,k\}}$ be the number of individuals with TB status i; where $i \in \{\text{Uninfected}, \text{Early LTBI}, \text{Late LTBI}, \text{Asymptomatic Active TB}, \text{Symptomatic Active TB}, \text{Recovered}\}$, living in setting j; where $j \in \{\text{high-risk}, \text{low-risk}\}$; and in age group k; where $k \in \{0-14, 15+\}$.

The following set of ordinary differential equations describe the model. The model parameters are described in Table S-1.

Uninfected:

$$\frac{\mathrm{d}X_{\{i=\text{Uninfected},j=\text{high-risk},k=0-14\}}}{\mathrm{d}t} = b\sum_i \sum_k X_{\{i,j=\text{high-risk},k\}} - [\mu_y + \lambda_h + 1/15]\, X_{\{i=\text{Uninfected},j=\text{high-risk},k=0-14\}}$$

$$\frac{\mathrm{d}X_{\{i=\text{Uninfected},j=\text{high-risk},k=15+\}}}{\mathrm{d}t} = -[\mu_o + \lambda_h]\, X_{\{i=\text{Uninfected},j=\text{high-risk},k=15+\}} + 1/15\, X_{\{i=\text{Uninfected},j=\text{high-risk},k=0-14\}}$$

$$\frac{\mathrm{d}X_{\{i=\text{Uninfected},j=\text{low-risk},k=0-14\}}}{\mathrm{d}t} = b\sum_i \sum_k X_{\{i,j=\text{low-risk},k\}} - [\mu_y + \lambda_l + 1/15]\, X_{\{i=\text{Uninfected},j=\text{low-risk},k=0-14\}}$$

$$\frac{\mathrm{d}X_{\{i=\text{Uninfected},j=\text{low-risk},k=15+\}}}{\mathrm{d}t} = -[\mu_o + \lambda_l]\, X_{\{i=\text{Uninfected},j=\text{low-risk},k=15+\}} + 1/15\, X_{\{i=\text{Uninfected},j=\text{low-risk},k=0-14\}}$$

Early LTBI:

$$\frac{\mathrm{d}X_{\{i=\text{Early LTBI},j=\text{high-risk},k=0-14\}}}{\mathrm{d}t} = \lambda_h\, X_{\{i=\text{Uninfected},j=\text{high-risk},k=0-14\}} - [\mu_y + s + p_y + 1/15]\, X_{\{i=\text{Early LTBI},j=\text{high-risk},k=0-14\}} + \xi\,\lambda_h \sum_{i\in\{\text{Late LTBI},\text{Recovered}\}} X_{\{i,j=\text{high-risk},k=0-14\}}$$

$$\frac{\mathrm{d}X_{\{i=\text{Early LTBI},j=\text{high-risk},k=15+\}}}{\mathrm{d}t} = \lambda_h\, X_{\{i=\text{Uninfected},j=\text{high-risk},k=15+\}} + 1/15\, X_{\{i=\text{Early LTBI},j=\text{high-risk},k=0-14\}} - [\mu_o + s + p_o]\, X_{\{i=\text{Early LTBI},j=\text{high-risk},k=15+\}} + \xi\,\lambda_h \sum_{i\in\{\text{Late LTBI},\text{Recovered}\}} X_{\{i,j=\text{high-risk},k=15+\}}$$

$$\frac{\mathrm{d}X_{\{i=\text{Early LTBI},j=\text{low-risk},k=0-14\}}}{\mathrm{d}t} = \lambda_l\, X_{\{i=\text{Uninfected},j=\text{low-risk},k=0-14\}} - [\mu_y + s + p_y + 1/15]\, X_{\{i=\text{Early LTBI},j=\text{low-risk},k=0-14\}} + \xi\,\lambda_l \sum_{i\in\{\text{Late LTBI},\text{Recovered}\}} X_{\{i,j=\text{low-risk},k=0-14\}}$$

$$\frac{\mathrm{d}X_{\{i=\text{Early LTBI},j=\text{low-risk},k=15+\}}}{\mathrm{d}t} = \lambda_l\, X_{\{i=\text{Uninfected},j=\text{low-risk},k=15+\}} + 1/15\, X_{\{i=\text{Early LTBI},j=\text{low-risk},k=0-14\}} - [\mu_o + s + p_o]\, X_{\{i=\text{Early LTBI},j=\text{low-risk},k=15+\}} + \xi\,\lambda_l \sum_{i\in\{\text{Late LTBI},\text{Recovered}\}} X_{\{i,j=\text{low-risk},k=15+\}}$$

Late LTBI:

$$\frac{\mathrm{d}X_{\{i=\text{Late LTBI},j=\text{high-risk},k=0-14\}}}{\mathrm{d}t} = s\, X_{\{i=\text{Early LTBI},j=\text{high-risk},k=0-14\}} - [\mu_y + \phi_y + \xi\,\lambda_h + 1/15]\, X_{\{i=\text{Late LTBI},j=\text{high-risk},k=0-14\}}$$

$$\frac{\mathrm{d}X_{\{i=\text{Late LTBI},j=\text{high-risk},k=15+\}}}{\mathrm{d}t} = s\, X_{\{i=\text{Early LTBI},j=\text{high-risk},k=15+\}} + 1/15\, X_{\{i=\text{Late LTBI},j=\text{high-risk},k=0-14\}} - [\mu_o + \phi_o + \xi\,\lambda_h]\, X_{\{i=\text{Late LTBI},j=\text{high-risk},k=15+\}}$$

$$\frac{\mathrm{d}X_{\{i=\text{Late LTBI},j=\text{low-risk},k=0-14\}}}{\mathrm{d}t} = s\, X_{\{i=\text{Early LTBI},j=\text{low-risk},k=0-14\}} - [\mu_y + \phi_y + \xi\,\lambda_l + 1/15]\, X_{\{i=\text{Late LTBI},j=\text{low-risk},k=0-14\}}$$

$$\frac{\mathrm{d}X_{\{i=\text{Late LTBI},j=\text{low-risk},k=15+\}}}{\mathrm{d}t} = s\, X_{\{i=\text{Early LTBI},j=\text{low-risk},k=15+\}} + 1/15\, X_{\{i=\text{Late LTBI},j=\text{low-risk},k=0-14\}} - [\mu_o + \phi_o + \xi\,\lambda_l]\, X_{\{i=\text{Late LTBI},j=\text{low-risk},k=15+\}}$$

Asymptomatic Active TB:

$$\frac{\mathrm{d}X_{\{i=\text{Asymptomatic Active TB},j=\text{high-risk},k=0-14\}}}{\mathrm{d}t} = \phi_y\, X_{\{i=\text{Late LTBI},j=\text{high-risk},k=0-14\}} + p_y\, X_{\{i=\text{Early LTBI},j=\text{high-risk},k=0-14\}} + r_2\, X_{\{i=\text{Symptomatic Active TB},j=\text{high-risk},k=0-14\}} - [\mu_{AT} + r_1 + \mathsf{w} + 1/15]\, X_{\{i=\text{Asymptomatic Active TB},j=\text{high-risk},k=0-14\}}$$

$$\frac{\mathrm{d}X_{\{i=\text{Asyptomatic Active TB},j=\text{high-risk},k=15+\}}}{\mathrm{d}t} = \phi_o\, X_{\{i=\text{Late LTBI},j=\text{high-risk},k=15+\}} + p_o\, X_{\{i=\text{Early LTBI},j=\text{high-risk},k=15+\}} + r_2\, X_{\{i=\text{Symptomatic Active TB},j=\text{high-risk},k=15+\}} + 1/15\, X_{\{i=\text{Asymptomatic Active TB},j=\text{high-risk},k=0-14\}} - [\mu_{AT} + r_1 + \mathsf{w}]\, X_{\{i=\text{Asymptomatic Active TB},j=\text{high-risk},k=15+\}}$$

$$\frac{\mathrm{d}X_{\{i=\text{Asymptomatic Active TB},j=\text{low-risk},k=0-14\}}}{\mathrm{d}t} = \phi_y\, X_{\{i=\text{Late LTBI},j=\text{low-risk},k=0-14\}} + p_y\, X_{\{i=\text{Early LTBI},j=\text{low-risk},k=0-14\}} + r_2\, X_{\{i=\text{Symptomatic Active TB},j=\text{low-risk},k=0-14\}} - [\mu_{AT} + r_1 + \mathsf{w} + 1/15]\, X_{\{i=\text{Asyptomatic Active TB},j=\text{low-risk},k=0-14\}}$$

$$\frac{\mathrm{d}X_{\{i=\text{Asymptomatic Active TB},j=\text{low-risk},k=15+\}}}{\mathrm{d}t} = \phi_o\, X_{\{i=\text{Late LTBI},j=\text{low-risk},k=15+\}} + p_o\, X_{\{i=\text{Early LTBI},j=\text{low-risk},k=15+\}} + r_2\, X_{\{i=\text{Symptomatic Active TB},j=\text{low-risk},k=15+\}} + 1/15\, X_{\{i=\text{Asymptomatic Active TB},j=\text{low-risk},k=0-14\}} - [\mu_{AT} + r_1 + \mathsf{w}]\, X_{\{i=\text{Asyptomatic Active TB},j=\text{low-risk},k=15+\}}$$

Symptomatic Active TB:

$$\frac{\mathrm{d}X_{\{i=\text{Symptomatic Active TB},j=\text{high-risk},k=0-14\}}}{\mathrm{d}t} = r_1\, X_{\{i=\text{Asymptomatic Active TB},j=\text{high-risk},k=0-14\}} - [\mu_{ST} + \omega_h\, k_h + r_2 + 1/15]\, X_{\{i=\text{Symptomatic Active TB},j=\text{high-risk},k=0-14\}}$$

$$\frac{\mathrm{d}X_{\{i=\text{Symptomatic Active TB},j=\text{high-risk},k=15+\}}}{\mathrm{d}t} = r_1\, X_{\{i=\text{Asymptomatic Active TB},j=\text{high-risk},k=15+\}} + 1/15\, X_{\{i=\text{Symptomatic Active TB},j=\text{high-risk},k=0-14\}} - [\mu_{ST} + \omega_h\, k_h + r_2]\, X_{\{i=\text{Symptomatic Active TB},j=\text{high-risk},k=15+\}}$$

$$\frac{\mathrm{d}X_{\{i=\text{Symptomatic Active TB},j=\text{low-risk},k=0-14\}}}{\mathrm{d}t} = r_1\, X_{\{i=\text{Asymptomatic Active TB},j=\text{low-risk},k=0-14\}} - [\mu_{ST} + \omega_l\, k_l + r_2 + 1/15]\, X_{\{i=\text{Symptomatic Active TB},j=\text{low-risk},k=0-14\}}$$

$$\frac{\mathrm{d}X_{\{i=\text{Symptomatic Active TB},j=\text{low-risk},k=15+\}}}{\mathrm{d}t} = r_1\, X_{\{i=\text{Asymptomatic Active TB},j=\text{low-risk},k=15+\}} + 1/15\, X_{\{i=\text{Symptomatic Active TB},j=\text{low-risk},k=0-14\}} - [\mu_{ST} + \omega_l\, k_l + r_2]\, X_{\{i=\text{Symptomatic Active TB},j=\text{low-risk},k=15+\}}$$

Recovered:

$$\frac{\mathrm{d}X_{\{i=\text{Recovered},j=\text{high-risk},k=0-14\}}}{\mathrm{d}t} = \omega_h\, k_h\, X_{\{i=\text{Symptomatic Active TB},j=\text{high-risk},k=0-14\}} + \mathsf{w}\, X_{\{i=\text{Asymptomatic Active TB},j=\text{high-risk},k=0-14\}} - [\mu_y + \xi\,\lambda_h + 1/15]\, X_{\{i=\text{Recovered},j=\text{high-risk},k=0-14\}}$$

$$\frac{\mathrm{d}X_{\{i=\text{Recovered},j=\text{high-risk},k=15+\}}}{\mathrm{d}t} = \omega_h\, k_h\, X_{\{i=\text{Symptomatic Active TB},j=\text{high-risk},k=15+\}} + \mathsf{w}\, X_{\{i=\text{Asymptomatic Active TB},j=\text{high-risk},k=15+\}} + 1/15\, X_{\{i=\text{Recovered},j=\text{high-risk},k=0-14\}} - [\mu_o + \xi\,\lambda_h]\, X_{\{i=\text{Recovered},j=\text{high-risk},k=15+\}}$$

$$\frac{\mathrm{d}X_{\{i=\text{Recovered},j=\text{low-risk},k=0-14\}}}{\mathrm{d}t} = \omega_l\, k_l\, X_{\{i=\text{Symptomatic Active TB},j=\text{low-risk},k=0-14\}} + \mathsf{w}\, X_{\{i=\text{Asymptomatic Active TB},j=\text{low-risk},k=0-14\}} - [\mu_y + \xi\,\lambda_l + 1/15]\, X_{\{i=\text{Recovered},j=\text{low-risk},k=0-14\}}$$

$$\frac{\mathrm{d}X_{\{i=\text{Recovered},j=\text{low-risk},k=15+\}}}{\mathrm{d}t} = \omega_l\, k_l\, X_{\{i=\text{Symptomatic Active TB},j=\text{low-risk},k=15+\}} + \mathsf{w}\, X_{\{i=\text{Asymptomatic Active TB},j=\text{low-risk},k=15+\}} + 1/15\, X_{\{i=\text{Recovered},j=\text{low-risk},k=0-14\}} - [\mu_o + \xi\,\lambda_l]\, X_{\{i=\text{Recovered},j=\text{low-risk},k=15+\}}$$

The forces of infection that individuals are subject to in the high- and low-risk settings are given by the following equations.

$$\lambda_h = \frac{\beta\,\exp(-(t-t_0)\,\beta_\Delta) * (\mathsf{T}_h + \sigma\,\mathsf{T}_l)}{N_h + \sigma\,N_l}$$

$$\lambda_l = \frac{\beta\,\exp(-(t-t_0)\,\beta_\Delta) * (\mathsf{T}_l + \sigma\,\mathsf{T}_h)}{N_l + \sigma\,N_h}$$

Here, β is the baseline transmission rate at time t_0 (reference: symptomatic adults in the high-risk population in the year 2000), β_Δ the rate of declines in transmission rate, N_h and N_l are the population size of high- and low-risk populations, respectively, σ is the mixing rate between the two populations, and T_h and T_l are transmission potential generated from high- and low-risk populations, respectively, as described below:

high-risk:

$$\mathsf{T}_h = \beta_y\beta_A\, X_{\{i=\text{Asymptomatic Active TB},j=\text{high-risk},k=0-14\}} + \beta_y\, X_{\{i=\text{Symptomatic Active TB},j=\text{high-risk},k=0-14\}} + \beta_A\, X_{\{i=\text{Asymptomatic Active TB},j=\text{high-risk},k=15+\}} + X_{\{i=\text{Symptomatic Active TB},j=\text{high-risk},k=15+\}}$$

low-risk:

$$\mathsf{T}_l = \beta_l[\beta_y\beta_A\, X_{\{i=\text{Asymptomatic Active TB},j=\text{low-risk},k=0-14\}} + \beta_y\, X_{\{i=\text{Symptomatic Active TB},j=\text{low-risk},k=0-14\}} + \beta_A\, X_{\{i=\text{Asymptomatic Active TB},j=\text{low-risk},k=15+\}} + X_{\{i=\text{Symptomatic Active TB},j=\text{low-risk},k=15+\}}]$$

Table S-1. Model parameters

Model parameter	Symbol	Parameter value or calibrated distribution (median; and 95% range)	Source or method of fitting/calibration
Per capita mortality rate among children, per year	μ_y	0.013	Taken to reflect age-distribution in India, with 27% under 15 years [20].
Per capita mortality rate among adults, per year	μ_o	0.018	Taken to reflect age-distribution in India, with 27% under 15 years [20].
Per capita birth rate	b	0.0197	Population Pyramids of the World from 1950 to 2100 [20]].
Per capita mortality rate among individuals infected with asymptomatic TB	μ_{AT}	0.025 (0.001 - 0.049)	Calibrated with initial samples drawn from the following distribution: $\mu_{AT} \sim$ unif$(0, 0.05)$
Per capita mortality rate among individuals infected with symptomatic TB	μ_{ST}	0.27 (0.17 - 0.42)	Calibrated with initial samples drawn from the following distribution: $\mu_{ST} \sim$ unif$(0.15, 0.6)$ [21].
Baseline *per capita* transmission rates (per infectious person-year)	β	28.7 (19.1 - 34.6)	Calibrated with initial samples drawn from the following distribution: $\beta \sim$ unif$(10, 35)$
Year-on-year decline in transmission rate	β_Δ	0.025 (0.017 - 0.034)	Calibrated with initial samples drawn from the following distribution: $\beta_\Delta \sim$ unif$(0.015, 0.035)$
Relative transmission, low-risk compared to high-risk	β_l	0.52 (0.27 - 0.96)	Calibrated with initial samples drawn from the following distribution: $\beta_\Delta \sim$ unif$(0.25, 1)$
Relative infectivity, children compared to adults	β_y	0.1 (0.003 - 0.2)	Calibrated with initial samples drawn from the following distribution: $\beta_y \sim$ unif$(0, 0.2)$
Relative infectivity, asymptomatic compared to symptomatic	β_A	0.23 (0.017 - 0.39)	Calibrated with initial samples drawn from the following distribution: $\beta_A \sim$ unif$(0, 0.4)$
Relative susceptibility due to immunologic protection among those with prior exposure (reinfection) compared to those without	ξ	0.39 (0.3 - 0.49)	Calibrated with initial samples drawn from the following distribution: $\xi \sim$ unif$(0.3, 0.5)$ [22,23,24]
Mixing; percentage of shared contacts between hotspot & general population	σ	5%	Assumed but previously analysed [25]
Rate of early progression among children, per year	p_y	0.024 (0.012 - 0.038)	Calibrated with initial samples drawn from the following distribution: $p_y \sim$ unif$(0.01, 0.04)$ [26]
Rate of early progression among adults, per year	p_o	0.045 (0.031 - 0.059)	Calibrated with initial samples drawn from the following distribution: $p_o \sim$ unif$(0.03, 0.06)$ [26]
Rate of late progression among children, per year	ϕ_y	0.0018 (0.00058 - 0.0029)	Calibrated with initial samples drawn from the following distribution: $p_y \sim$ unif$(0.0005, 0.003)$ [27,28]
Rate of late progression among adults, per year	ϕ_o	0.0027 (0.0011 - 0.0048)	Calibrated with initial samples drawn from the following distribution: $p_o \sim$ unif$(0.001, 0.005)$ [27,28]
Average time to stabilization	$\frac{1}{s}$	5 years	Assumed, but in line with previous work [24]. We also considered a shorter duration of 2 years in Fig. S-5

Rate of progression from asymptomatic to symptomatic, per year	r_1	2.6 (1.1 - 3.9)	Calibrated with initial samples drawn from the following distribution: $r_1 \sim$ unif$(1, 4)$
Rate of regression from symptomatic to asymptomatic, per year	r_2	$r_1 - r_2$ distribution: 0.55 (0.027 - 0.98)	Calibrated with initial samples drawn from the following distribution: $r_1 - r_2 \sim$ unif$(0, 1)$
Rate of spontaneous resolution, per year	w	0.68 (0.19 - 0.98)	Calibrated with initial samples drawn from the following distribution: w $\sim$ unif$(0.1, 1)$ [29]
Rate of diagnosis, low-risk population	ω_l	1.69 (0.96 - 1.99)	Calibrated with initial samples drawn from the following distribution: $\omega_l \sim$ unif$(0.5, 2)$ in line with WHO estimates [1].
Rate of diagnosis, high-risk population	ω_h	0.58 (0.32 - 1.2)	Calibrated with initial samples drawn from the following distribution: $\omega_l \sim$ unif$(0.3, 1.3)$in line with WHO estimates [1].
Proportion cured, low-risk population	k_l	0.92 (0.87 - 0.97)	Calibrated with initial samples drawn from the following distribution: $k_l \sim$ unif$(0.87, 0.97)$in line with WHO estimates accounting for relapses after apparent treatment success and for cures after loss to follow up [1,30].
Proportion cured, high-risk population	k_h	0.87 (0.82 - 0.92)	Calibrated with initial samples drawn from the following distribution: $k_l \sim$ unif$(0.82, 0.92)$in line with WHO estimates accounting for relapses after apparent treatment success and for cures after loss to follow up [1,30].

S-2 Model calibration

The calibration process aimed to capture key demographic and epidemiological features of TB in the urban Indian setting. We considered seven demographic and epidemiological measures for model calibration and identified a data-consistent target range for each measure, as listed in Table 1 in the main text. To calibrate the model, we first used Latin Hypercube Sampling to sample 500,000 sets of model parameter values describing TB natural history and standard of care, from ranges shown in Table S-1. For each parameter set, we simulated the model for 520 years; the first 500 years were used to bring the model to equilibrium, and the final 20 years' worth of simulations, representing the time period from 2000 to 2020, were recorded for model calibration. We assumed that transmission rate was fixed (i.e., no decline in transmission rate) for the first 500 years of simulation. For the final 20 years, we allowed transmission rate to decline (i.e., $\beta_\Delta \geq 0$), to capture decline in TB incidence. Simulations which yielded model outputs that were consistent with all calibration targets were selected, such that the calibrated model consisted of equally weighted samples of simulations in which all of the model outputs considered for calibration were within their respective calibration target ranges. Simulations of the calibrated model are provided in Fig S-1.

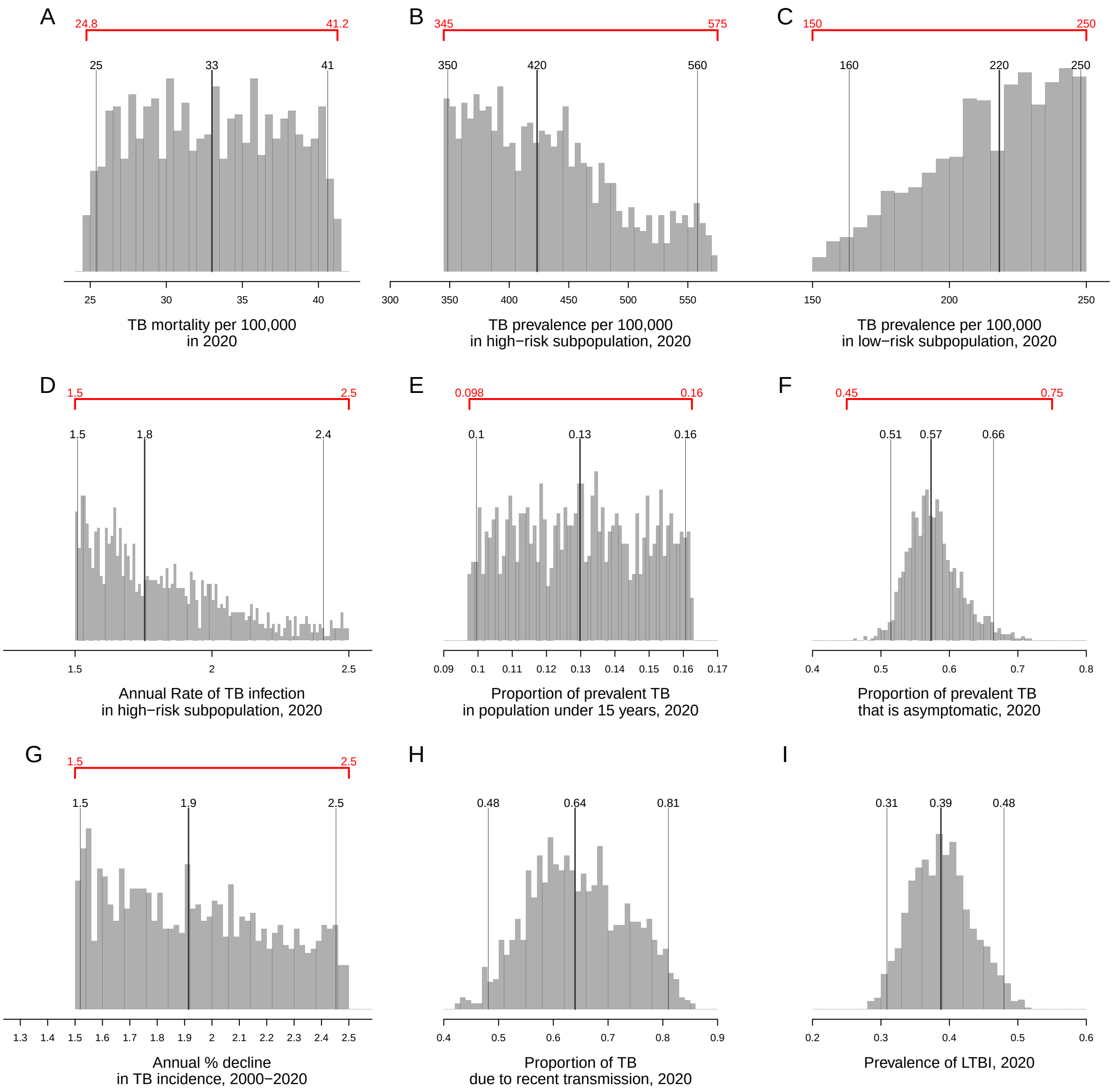

Figure S-1: Comparison of model simulations, and calibration targets. Shown are distributions of various model outcomes in simulations of the calibrated models. The vertical lines indicate median and 2.5^{th} and 97.5^{th} percentile values. For outcomes which were considered for model calibration, the red lines indicate respective calibration targets.

S-3 Modeling the effects of the one-time intervention

The one-time intervention was modeled as moving specified proportions of each latent or active TB compartment to the corresponding recovered state. The proportions were estimated as a product of proportions who would receive or successfully complete each step of the intervention, as follows:

Table S-2. Model parameter for one-time intervention

Model parameter/step of intervention	Estimate	Source or rationale
Proportion of adults contacted during intervention and completing chest x-ray (CXR) and tuberculin skin test (TST) placement	70% overall (differs by subpopulation in sensitivity analysis)	Estimation of reasonable coverage by implementation partners
Proportion of active TB detected by chest x-ray and confirmed by sputum Xpert Ultra	Adults, symptomatic TB: $= (0.95 \times 0.88) = 83.6\%$ Adults, asymptomatic TB: $= (0.95 \times 0.7) = 66.5\%$ Children, symptomatic TB: $= (0.95 \times 0.7) = 66.5\%$ Children, asymptomatic TB: $= (0.95 \times 0.7 \times 0.8) = 53.2\%$	For adults, product of 95% sensitivity of chest x-ray for culture-positive TB in prevalence surveys [34] (assuming for this model that TB with no CXR abnormalities or symptoms is epidemiologically inconsequential) and 88% sensitivity of Xpert Ultra for symptomatic TB relative to multiple cultures [35] (reduced to 70% if asymptomatic, based on higher prevalence of smear-negative disease). For children, reduced 20% based on lower sensitivity of sputum diagnosis in children [36].
Proportion initiating treatment, if Xpert positive	91%	Assumed same as for TPT (below)
Proportion cured by treatment, if treatment initiated through intervention	90%	Assumes public sector treatment initiation, and that some of those who are lost to follow up or not evaluated in programmatic outcome data are cured by partial treatment [1]. TPT was assumed to have no impact on the course of active TB that was not detected by screening.
Proportion completing TST reading, if TST placed	89%	Based on experience with population-wide LTBI intervention in Majuro, Marshall Islands
Sensitivity of TST (proportion TST positive, if LTBI)	90%	[37,31,32]. Assuming 10mm cutoff.
Proportion initiating TPT, if TST positive (and negative for active TB)	91%	Based on Majuro data, where of TST+, 2% were ineligible for TPT and 7% declined (unpublished).
Proportion completing TPT, if initiated	86%	Based on experience in Majuro
Proportion of future TB reactivations prevented, if TPT completed	69%	Derived from 3HP efficacy estimate from a network metaanalysis: odds ratio 0.36, at cumulative incidence $<$10% $\rightarrow$ risk ratio 0.38 $\rightarrow$ 62% of TB prevented overall, in 90% adherent population $\rightarrow$ $.62/.9 = 69\%$ reduction in TB among those who completed therapy. [33]
Proportion of TB-affected children who are a contact of an adult with active TB (and thus eligible for the intervention)	10%, children with active TB 3%, children with early LTBI Varies ($<$1%), children with late LTBI	See below

Proportion of eligible children who initiate intervention (get TST placement if >5y, or TPT referral if <5y).		For those requiring TST, we use same reading rate and sensitivity as for adults. We also assume the same TPT uptake, completion, and efficacy as for adults. All child contacts <5y are referred for TPT.
Proportion of child contacts with active TB who are diagnosed with active TB		Treatment uptake and outcomes assumed same as for adults.

The proportion of children reached by the intervention was estimated by first estimating what fraction of children with a given TB status were close contacts of an adult with current active tuberculosis that could potentially be identified by the intervention, and then multiplying this by the proportion of adults with active TB whose TB was detected by the intervention (regardless of whether the adult initiated or completed treatment).

We first assumed that 20% of TB transmission to children occurs without households [38]. Then, for children with current active TB, we assumed that 50% had acquired their TB infection from an index case who still had undiagnosed active TB, and that the remainder had been infected by someone who was already treated, resolved, or deceased at the time of the adult case-finding intervention. This resulted in an estimate that 10% of children with active TB could potentially be reached by the intervention.

For children with early LTBI, we reduced the proportion with a currently-active case to 15%, based on the longer (5 year) modeled duration of early LTBI relative to the duration of active TB, such that most index cases would no longer have active disease. This resulted in an estimate that 3% of children with early LTBI could potentially be reached by the intervention. Finally, for children with late latent LTBI, we assumed that the prevalence of active TB among their adult contacts was equal to the overall prevalence of active TB among adults in that child's subpopulation (high-risk or low-risk subpopulation). We estimated that each child had close contact with an average of 2 adults, such that the probability that a child had contact with an adult case was equal to twice the prevalence of active TB among adults. This probability was estimated at the time of the intervention for each subpopulation.

S-4 Modeling the effects of medium-term health system strengthening

Our compartmental transmission model included only a treatment rate parameter (ω) that applied to all symptomatic TB, and a treatment success probability (k) for those who initiated treatment.

We conceptualized ω as consisting of multiple components: an average time to care-seeking once symptomatic (t_1), an average time to diagnosis and initiation of treatment (t_2), and a probability of pretreatment loss to follow-up (p). Thus, $\omega = (1-p)/(t_1+t_2)$.

We estimated a value of 1 month for t_2 [39,40,41] and 16% for p [42]. We modeled health system strengthening as reducing each of the following by a factor m:

- Time to seek care once symptomatic (t_1)
- Time to diagnosis and treatment initiation (t_2)
- Probability of pretreatment loss to follow up (p)
- Probability of poor treatment outcomes ($1-k$)

Solving for t_1 in terms of ω, p, and t_2, and applying factor m to each of t_1, t_2, p, and $1-k$, we modified the k and ω parameters as follows:

$$k' = 1 - m(1-k)$$

and

$$t_1' = m\,[(1-p)/\omega - t_2]$$

$$\omega' = (1-p')/(t_1'+t_2') = \frac{1-m\,p}{m\,[(1-p)/\omega - t_2] + m\,t_2} = \frac{(1-mp)\omega}{m\,(1-p)}$$

S-5 Sensitivity analyses

S-5.1 Sensitivity analyses of TB cases averted

We examined impact of variation in model parameters to the secondary outcome, TB cases averted over 10 years by a combined intervention of a one-time campaign plus health system strengthening. As shown in Fig. S-2, the median TB cases averted per 1 million corresponding to parameter values in the top and bottom deciles differed by more than 1,950 (25% of median) for only two of the modeled parameters: (i) reactivation rate for adults (where the difference in the outcomes corresponding to top and bottom deciles was 2,015 cases), and (ii) rate of spontaneous resolution (2,033 cases). This indicates that when (i) LTBI cases have higher expected lifetime risk of reactivating, and (ii) a higher proportion of TB cases are not diagnosed through standard TB care, the intervention is likely to be more impactful in averting potential TB cases.

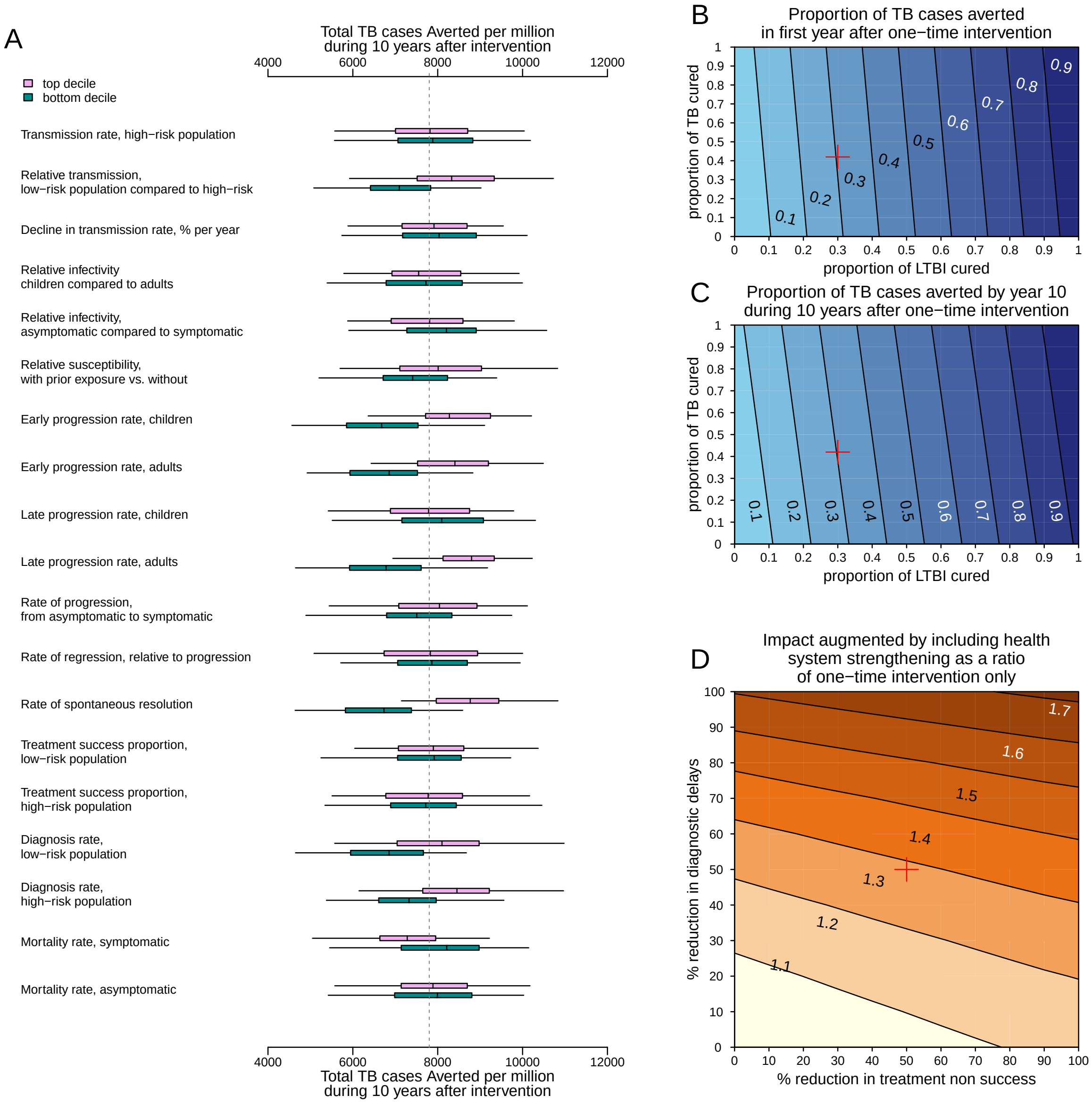

Figure S-2: Sensitivity analyses of the secondary outcome, TB cases averted over 10 years by a combined intervention of a one-time campaign plus health system strengthening. (A) Sensitivity of the secondary outcome (TB-related cases averted over 10 years by a combined intervention of a one-time campaign plus health system strengthening), to individual model parameters. Each pair of boxplots shows variation in the outcome when analysis was limited to either simulations in which the value of the parameter of interest was in the top (light pink) or bottom (dark green) decile of its values across all accepted simulations. In each boxplot, the edges of the box represent the lower and upper interquartile range, the band in the middle represents the median, and the end of the whiskers represent 2.5^{th} and 97.5^{th} percentiles. The vertical dotted line shows the median across all accepted simulations. (B-C) Contours show the proportion of TB deaths averted by year 1 (B) and by year 10 (C) after a one-time campaign (with no subsequent health system strengthening) that achieves cure of LTBI in the proportion of the population indicated on the x axis and cure of active TB in the proportion indicated on the y axis. (D) Colored level-surfaces indicate additional impact on TB incidence of including health system strengthening measures with a one-time campaign, relative to the impact of the one-time campaign alone, assuming 70% coverage with one-time intervention, and a specified percentage reduction in unsuccessful treatments (x-axis) and diagnostic delays (y-axis). Red cross in panels B-D indicates the reference scenario.

S-5.2 Comparing the impact of curing LTBI versus TB disease

We compared the impact of active case finding and preventive therapy, by comparing the number of cases averted by successfully treating one case of LTBI vs. one case of TB. The number of cases averted by successfully treating one case of LTBI was estimated by dividing the total number of cases averted through preventive therapy (shown in Fig. 3, blue lines) by the total number of individuals successfully treated for LTBI as a result of preventive therapy campaign. We estimated that 30.3% of individuals with LTBI were cured through preventive therapy, as shown in Fig. 2, and the median LTBI prevalence was 39% (95% range: 31%-48%) as shown in Fig.S-1-I. Similarly, the number of cases averted by successfully treating one case of TB was estimated by dividing the number of cases averted through ACF (shown in Fig. 3, yellow lines) by the number of individuals successfully treated for TB disease as a part of ACF campaign. Approximately 40% of individuals with active TB disease was cured through ACF, as shown in Fig. 2, and the median prevalence of TB disease was 260 (95% range: 210–300) per 100,000, as shown in Fig. 1-A.

We note that the impact of treating LTBI is realized slowly over time, and is about 10-30 times smaller than that of treating TB in a per capita basis (Fig. S-3).

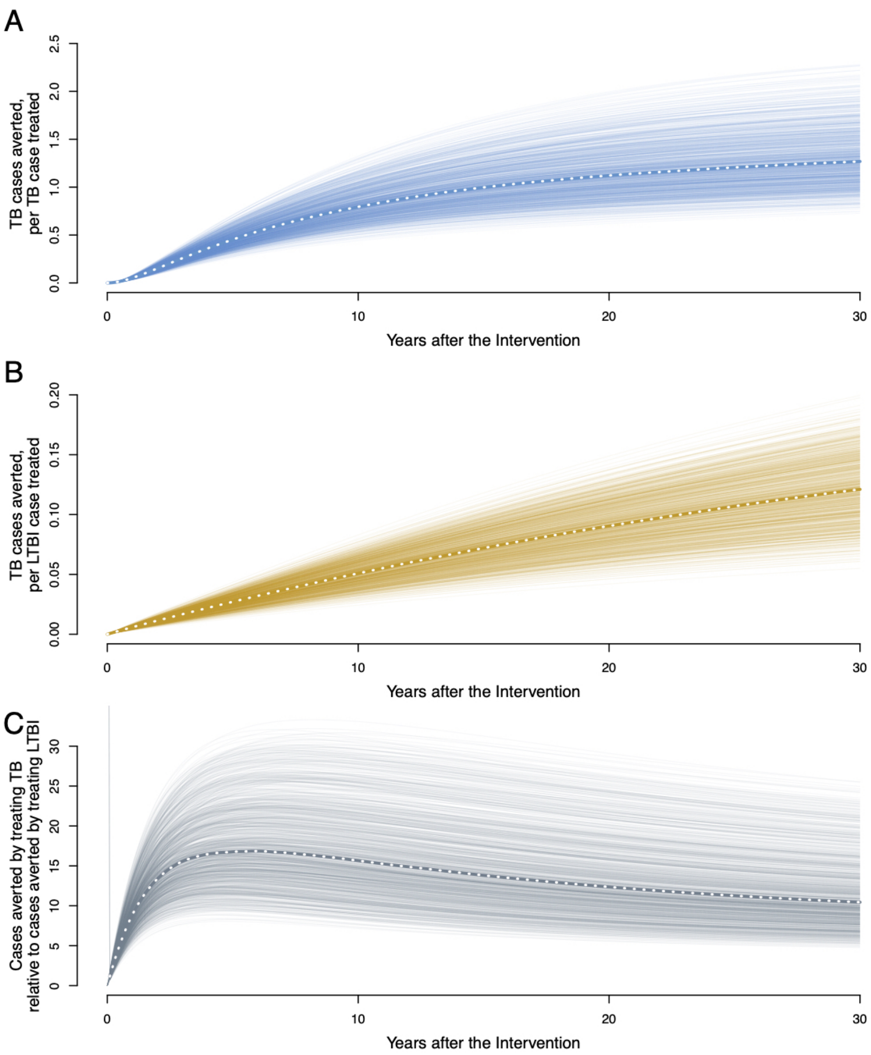

Figure S-3: Comparing the impact of curing LTBI versus TB disease. Shown are estimated number of TB cases averted over time (A) per LTBI case treated, and (B) per TB case treated. Resulting ratio between the two are shown in (C).

S-5.3 Targeting the one-time intervention

We explored the impact of targeting the intervention to the high-risk population. We considered a scenario in which individuals in the high-risk population were screened preferentially to those in the low-risk population, where the the odds ratio, i.e., ratio of the odds of screening in the high-risk population to the odds of screening in the low risk population, was 5:1. The impact of this targeted one-time intervention (without health system strengthening) was modestly larger compared to the untargeted scenario presented in the main text. The cumulative TB-related deaths averted after 10 years was 870 (655 - 1,090) compared to 809 (612 - 1,010); and the cumulative cases averted was 6,090 (4,310 - 7,850) compared to 5,840 (4,060 - 7,650) per 1 million population. (See Fig. S-4.)

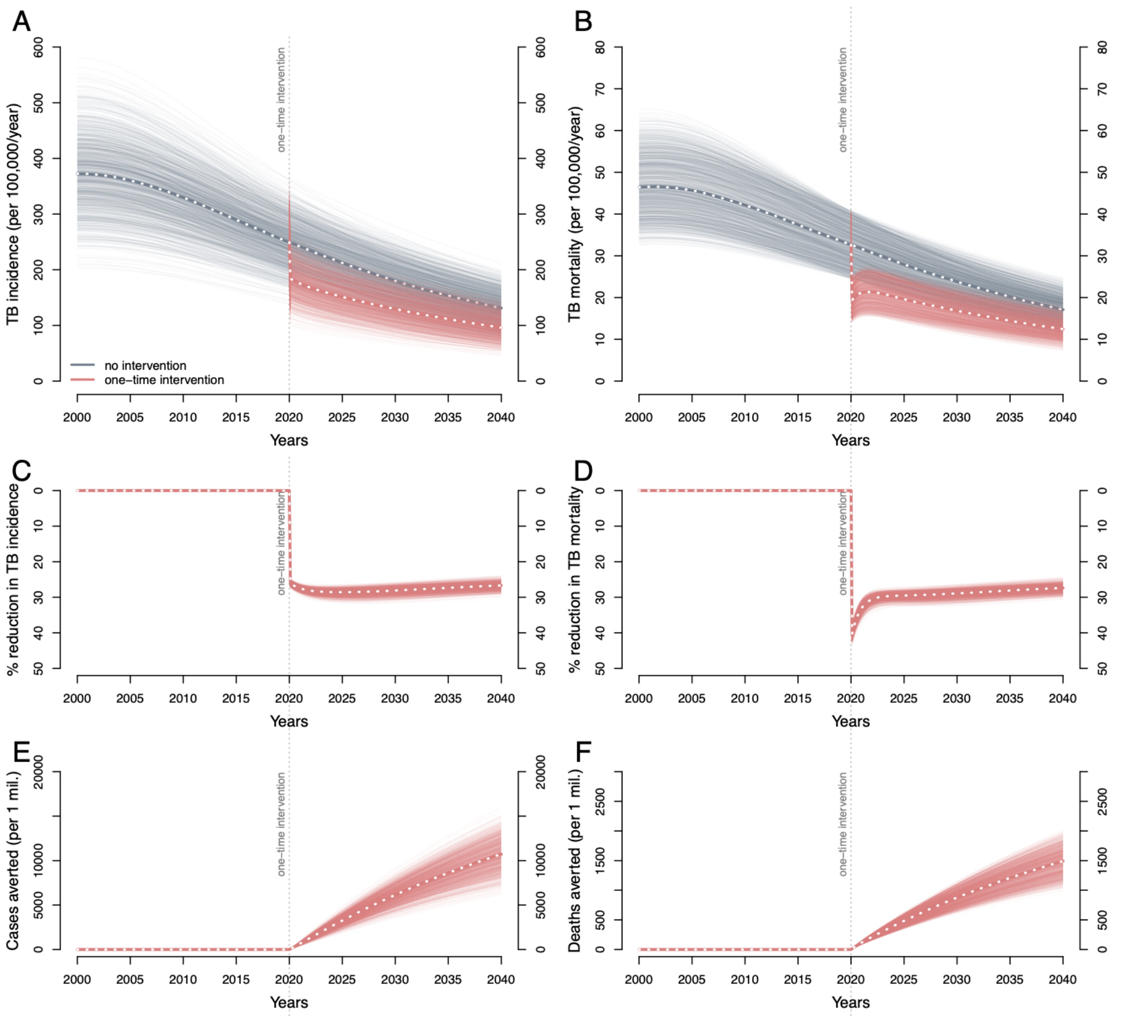

Figure S-4: The impact of a one-time intervention (without health system strengthening), when the intervention was targeted to the high-risk population. Shown in (A) and (B), respectively, are TB incidence rate and TB-related mortality rates, per 100,000 per year between 2000 to 2040, in model simulations without the intervention (grey), and the simulations with the intervention implemented in 2020 (red). Shown in (C) and (D) are percentage reductions in TB incidence and TB-related mortality rates, respectively. Shown in (E) and (F) are, respectively, cumulative number of TB cases and TB-related deaths averted per 1 mil. population.

S-5.4 Shorter Early LTBI

For the analyses in the main text, we assumed the average duration of early LTBI to be 5 years. Here, we assumed that the average duration to be only 2 years. We recalibrated the model, using the same procedure and calibration targets. We estimated the impact of one-time intervention (without health system strengthening) compared it to the reference scenario presented in the main text (See Fig. S-5). The estimated cumulative deaths averted after 10 years was 860 (649 - 1,070); and the estimated cumulative cases averted was 6,250 (4,320 - 8,050).

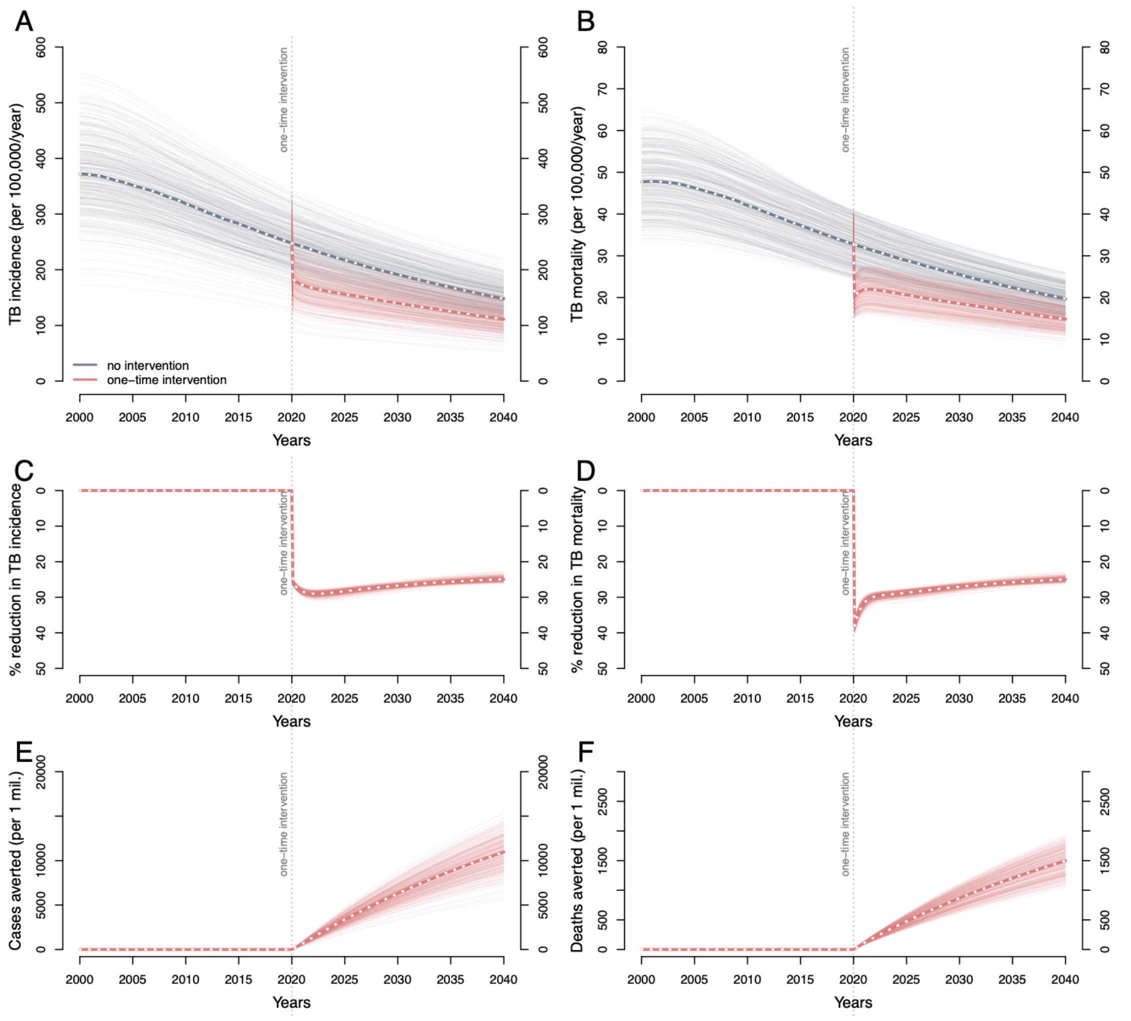

Figure S-5: The impact of a one-time intervention (without health system strengthening), with shorter duration of early LTBI. Shown in (A) and (B), respectively, are TB incidence rate and TB-related mortality rates, per 100,000 per year between 2000 to 2040, in model simulations without the intervention (grey), and the simulations with intervention implemented in 2020 (red). Shown in (C) and (D) are percentage reductions in TB incidence and TB-related mortality rates, respectively. Shown in (E) and (F) are, respectively, cumulative number of TB cases and TB-related deaths averted per 1 mil. population.

S-5.5 Preventive therapy targeted to recent infections

For these analyses, we assumed that as a part of the comprehensive intervention, preventive therapy was only provided to individuals in Early LTBI compartment, i.e., individuals with recent exposure. Compared to the full intervention in which preventive therapy is provided to all LTBI, this intervention resulted in lower impact: a median 35% less cases and 11% less deaths averted after 10 years of intervention. However, the number of individuals receiving preventive therapy during this targeted intervention was about one-tenth compared to the full intervention.

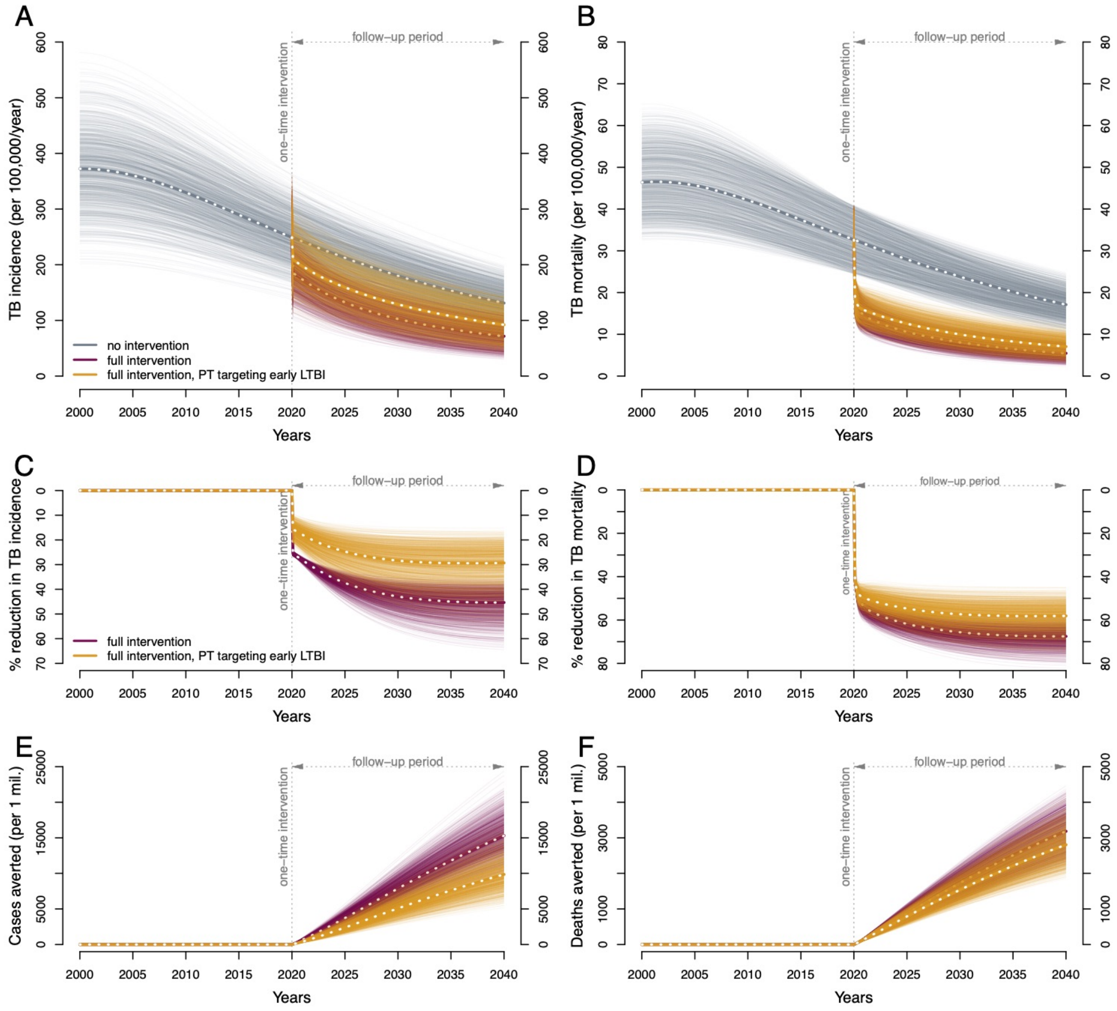

Figure S-6: The impact of the full intervention, when preventive therapy is limited to recent infections. Shown in (A) and (B), respectively, are TB incidence rate and TB-related mortality rates, per 100,000 per year between 2000 to 2040, in model simulations without the intervention (grey), and the simulations with intervention implemented in 2020 (red). Shown in (C) and (D) are percentage reductions in TB incidence and TB-related mortality rates, respectively. Shown in (E) and (F) are, respectively, cumulative number of TB cases and TB-related deaths averted per 1 mil. population.
